# Multicolor Emitting Carbon Dot-Reinforced PVA Composites as Edible Food Packaging Films and Coatings with Antimicrobial and UV-Blocking Properties

**DOI:** 10.1021/acsomega.2c02984

**Published:** 2022-08-22

**Authors:** Melis Özge Alaş, Gamze Doğan, Mustafa Serkan Yalcin, Sadin Ozdemir, Rükan Genç

**Affiliations:** † Department of Chemical Engineering, Engineering Faculty, Mersin University, Mersin TR-33343, Turkey; ‡ Faculty of Engineering Department of Bioengineering, Izmir Institute of Technology, Urla-Izmir TR-35430, Turkey; § Department of Chemistry and Chemical Processing Technologies, Technical Science Vocational School, Mersin University, Mersin TR-33343, Yenisehir, Turkey; ∥ Food Processing Programme, Technical Science Vocational School, Mersin University, Mersin TR-33343, Yenisehir, Turkey; ⊥ Nanotechnology Research and Application Centre, Sabanci University, Istanbul TR-34956, Turkey

## Abstract

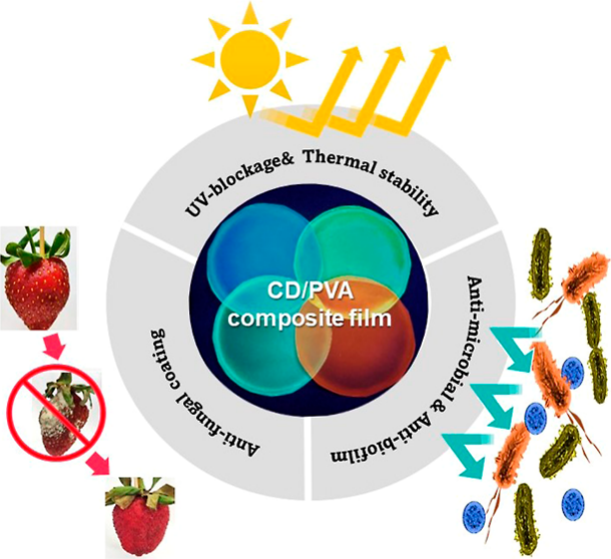

Active food packaging has become attractive because of the possibility to provide a longer shelf-life by loading functional agents into the packages to maintain the quality of food products. Herein, photoluminescent and transparent polyvinyl alcohol (PVA)-based composites embedding multicolor fluorescent carbon dots (CD/PVA) were prepared by the solvent casting method. The prepared CDs emit a strong and stable fluorescence in solution while the CD/PVA composite films were transparent, flexible, and showed UV-blocking activity with a strong fluorescence emission. Blue color-emitting CDs showed the highest UV blockage at UVA (87.04%), UVB (87.04%), and UVC (92.22%) regions while PVA alone absorbed only less than 25% of the light in all UV regions. UV blockage capacity was shown to be decreased by half, in line with the emission color shift from blue to red. Thermal properties of the PVA film were improved by the addition of CDs to the polymer, and in vitro cell viability tests showed that none of the CDs were cytotoxic against the human lung fibroblast healthy cell line (MRC-F cells) when integrated into the PVA. The antimicrobial activity of CD/PVA nanofilms was qualitatively determined. The prepared films exhibited good antimicrobial activity against both Gram-positive and Gram-negative bacteria with mild antioxidant and metal chelating activity, and significant inhibition of biofilm formation with a strong link with emitted color and the concentration of the composites. Green- and red-emitting CD/PVA with the highest antimicrobial activity were then analyzed and compared with the plane PVA employing their effect on the shelf-life of strawberries as a model for perishable foods. Fresh strawberries dip coated with CD/PVA and PVA were monitored over time, and virtual evaluations showed that CDs/PVA film coating resulted in reduced weight and moisture loss and significantly inhibited the fungal growth and spoiling for over 6 days at RT and 12 days at fridge conditions maintaining the visual appearance and natural color of the fruit. The findings in this work indicated the potential of reported CD as non-cytotoxic, UV-blocking antimicrobial additives for the development of edible coatings and packages for their use in the food industry, as well as pharmaceutical and healthcare applications.

## 1 Introduction

Food waste reduction is an important part of developing a sustainable economy. Global food loss and waste are between one-third and half of all food produced each year due to shelf-life finishing or corruption owing to microbial activity. The Food and Agriculture Organization of the United Nations (FAO) in 2020 reported that around 14% of food produced for human consumption worldwide was lost before reaching the market.^1^ Perishable crops such as fresh fruits and vegetables with high metabolic activity might show a high risk of microbial contamination, which quickly spoils and results in color change, bad taste, and smell.^2,3^ Food packaging plays an important role in food safety and quality aiming for high food spoilage blocking and shelf-life improvement by protecting food from chemical and physical hazards, external pathogens, and microorganisms.^4^ Antimicrobial food packaging has become attractive because of the ability to stop or delay microbiological degradation and provide longer shelf life by loading antimicrobial (natural or synthetic) agents into the packages to maintain the quality and sensory properties of food products.^5,6^ In the last decade, antimicrobial edible films and coatings have been used in different food products and have been shown to preserve their integrity (apples, strawberry, grape berry, kiwi, carrots, cucumber, cabbage, meat, etc.) by preventing pathogenic microorganisms and spoilage caused by contamination and delaying enzymatic oxidation.^7−9^ Materials used in food coatings/films should be made up of biocompatible, sustainable, and biodegradable sources with UV shielding capability with antimicrobial and antioxidative properties. At the same time, mechanically stable matrices with hydrophobic groups (natural and synthetic polymers, etc.) should be used to provide low permeability to oxygen and moisture.^3,10^

In order to develop new food packaging technologies, polymer nanocomposites that maintain their recyclability and offer the desired functionalities (such as oxygen barrier, antimicrobial, antifungal, color, and appearance) can be used by combining the properties of a nanoscale filler with natural (chitosan, starch, cellulose, alginate, etc.) and synthetic polymers (polypropylene, polyethylene, polyvinyl chloride, polyvinyl alcohol, etc.) used in food packaging technologies.^11−16^ In addition, because the food contact materials are often exposed to solar UV light, UV-absorbing fillers are added to various polymeric materials such as polyimides, collagen, cellulose, PVA, and so forth to prevent degradation, thereby improving the UV shielding properties of food packages.^17−20^ Polyvinyl alcohol (PVA) is an edible, non-toxic, biodegradable, environmentally friendly synthetic polymer that was approved by the Food and Drug Administration (FDA) for clinical use in humans.^21,22^ PVA has been used as an additive component in the coating and packaging of food products due to these properties as well as its excellent oxygen barrier properties, good film-forming capacity, and high mechanical properties.^23,24^ However, PVA has difficulties such as poor thermal stability and biological activity and high moisture absorption, and it provides poor protection against UV exposure when used in food packaging systems. To improve these properties and add functionality to the polymer, organic and inorganic nanomaterials have been combined with PVA thanks to the presence of hydrogen bonding groups in the PVA structure, which facilitates the creation of functional nanocomposites with other nanomaterials.^25,26^ For example, Yu et al. have produced SiO_2_ in situ-enhanced PVA/chitosan biodegradable films and reported that these films reduce moisture permeability and oxygen permeability in food packages to maintain freshness.^27^ Salman and co-workers demonstrated the usability of PVA/nanocellulose/Ag nanocomposite films with strong mechanical and antibacterial properties as antimicrobial food packaging materials.^28^ Abdullah et al. have developed water-resistant, biodegradable, and acceptable transparent poly(vinyl) alcohol/starch/glycerol/halloysite nanotube nanocomposite films for sustainable food packaging of lipophilic and acidic foodstuffs.^29^ Moreira and co-workers have advanced a polysaccharide/PVA-based edible antimicrobial coating material as an environmentally friendly and bioactive food packaging material that prevents fungal growth and decreases water loss of fresh fruits during storage.^23^ Chowdhury et al. have produced poly(vinyl) alcohol cross-linked composite packaging films containing gold nanoparticles that extend the shelf life of bananas and exhibit antimicrobial activity in food packaging systems.^30^ As a result, they reported that composites prepared by integrating different nanomaterials had better functional properties than pure PVA.

Recently, carbon materials have been given great importance as nanofillers in the preparation of functional polymer nanocomposites to improve the properties of PVA films.^31,32^ Sapalidis et al. have produced PVA nanocomposite films containing dendritic polymer (QPEI)-functionalized multi-walled carbon nanotubes (oxCNTs) using various oxCNT QPEI contents ranging from 0.05 to 1.0% w/w and reported that these films exhibited enough optical transparency, advanced mechanical properties, and exceptionally high antibacterial behavior.^31^ Kovalchuk and co-workers have produced luminescent PVA composite films containing coal-derived graphene quantum dots (GQDs) by a simple and environmentally friendly solution method. They report that PVA/GQD nanocomposites exhibit broad photoluminescence emission spectra covering most of the visible range and that these materials can be used as luminophores in white-light LEDs as an alternative to toxic conventional inorganic quantum dots.^33^ Kwan et al. produced composite films by immobilizing carbon dots (CDs) synthesized by the microwave-assisted hydrothermal method to PVA and PVA/PEG polymer matrixes. They used these films for the detection of tartrazine, a synthetic food dye, and reported that they can be used as a tartrazine sensor in food quality control, as they can detect tartrazine at a concentration as low as 10 μM.^34^

CDs attracted broad research interest for years due to their photophysical properties, non-toxicity, good solubility, high photoluminescence, high thermal stability, abundant functional groups (e.g., amino, hydroxyl, carboxyl), large surface area, and good biocompatibility,^35,36^ and there currently have been used in biosensors, supercapacitors, fingerprint information storage, solar cells, white light-emitting diodes (WLEDs), and optoelectronic devices.^37−41^ Various methods are available for CD syntheses such as arc discharge, laser ablation, electrochemical synthesis, and ultrasonic treatment.^42−45^ In addition, methods such as thermal, hydrothermal, and microwave syntheses to prepare CD from a large number of precursors are low price, fast, easy, cheaper, and environmentally friendly alternatives.^46,47^ Lately, some green synthetic approaches have become popular, using cheap, renewable, and environmentally friendly natural products to produce CDs as a carbon source (peanut shell, molasses, lemon salt, carrot, beverages, plants, etc.).^48−52^ CDs have good biocompatibility, low cytotoxicity, and an easily modifiable surface, as well as effective UV blocking, anti-inflammatory, antioxidant, and antimicrobial properties.^53−56^ It has become attractive to produce a new type of multifunctional nanoplatforms in which the optical, chemical, and biological properties of CDs are integrated into bio-compatible polymeric matrices. To date, very few papers exist on the role of these new CD/polymer nanocomposites in food packaging in the literature. Purkayastha et al. have produced rapeseed protein-based fluorescent films with CDs with antioxidant potential and thermal stability and examined the effect of rapeseed oil on oxidative shelf life. They reported that oil samples packaged in bags made of an FCD–protein composite film resisted oxidation better than those stored in intact protein-based bags.^57^ Patil and co-workers have produced an alternative UV blocker polyvinyl alcohol/waste tea residue carbon dot (PVA @ WTR-CD) composite film that is transparent, thin, flexible, re-emissive, and has high mechanical strength using sustainable and green synthesis methods. These composite films for the fruit packaging application have been tested on grapefruit and demonstrated that they have great potential as ready-made materials in the agricultural sector for safe food packaging applications.^58^ Kousheh et al. have predicted that the polymeric system, in which photoluminescent CDs obtained from probiotic bacteria are integrated, could be a new biomaterial for anti-microbial and UV-protective food active packaging, increasing its antimicrobial/antioxidant and UV protective properties.^59^ Zhang et al. have produced a fluorescent food packaging film (CA-CD-FF) by adding CDs to the PVA film to monitor food quality. They showed that CA-CC/PVA used as both film and coating can be used to alter the shelf life of bananas, determine food spoilage, and detect Al^3+^ and basic substances as contaminants in food samples.^60^ Although these studies strongly emphasize the UV blocking properties of CD-integrated food packaging systems, there is little research on their antimicrobial, antifungal, and UV protection properties for development of the edible films and coatings for protecting perishable fruits and vegetables.

Herein, we report an easy approach to produce a series of photoluminescent polymer composite films via a one-step method by in situ embedding multicolor fluorescence-emitting CDs in a PVA polymer matrix as edible and UV-protecting coating to extend the shelf life of the perishable foods like strawberries. The optical and thermal properties of the thin films were analyzed in detail and biological activities of CD/PVA nanocomposite films were pursued in order to fully explore their potential as UV-shielding antimicrobial food packaging and coating material and discussed employing emitted color and surface characteristics of the CDs. Their antifungal activity under different storage conditions was evaluated on strawberries as a highly perishable food model.

## 2 Materials and Methods

### 2.1 Materials

Polyethylene glycol (PEG) and urea were purchased from Sigma-Aldrich. Polyvinyl alcohol (PVA, average molecular weight: 31,000–50,000) was purchased from Acros Organics. Paraphenylenediamine (p-PD) was purchased from Alfa Aesar. Ethanol was purchased from J.T Baker. Lemon salt and carob molasses were obtained from a local supermarket. Ultrapure water used throughout all the experiments was purified by a Milli-Q system (Millipore Inc., Ω = 18 MΩ cm). Fresh strawberries were bought from the greengrocer (Mersin, Turkey).

### 2.2 Equipment

The optical properties of the prepared CD solution and nanocomposite films were recorded on a Shimadzu UV-1800 UV–vis spectrophotometer and Analytic Jena Specord 210 spectrometer in the range of 200–900 nm emission measurements of CD solutions and the nanocomposite films were carried out using a Varian Cary Eclipse fluorescence spectrophotometer and Shimadzu RF-5301 spectrofluorophotometer, respectively. The emission spectrum of all films except the YCD/PVA film (λ_exc_: 480 nm) was recorded at 365 nm excitation wavelength. Fluorescence images of CD solutions and nanocomposite films were taken using a UV Lamp (254/365 nm, UVP UVGL-58, Analytik Jena). ^1^H NMR measurements were performed in deuterated water (D_2_O) for BCD, GCD, and YCD and in deuterated DMSO for RCD using a 400 MHz Bruker NMR device. Transmission electron microscopy (TEM) (Model JEOL USA JEM-1400) was used to characterize the morphology and particle size of the synthesized CDs. In order to define the surface morphology of the obtained films, these were observed using field-emission scanning electron microscopy (FE-SEM, Zeiss Supra 55). The samples were dried by a critical point drying device and coated twice with platin. The functional groups present in the synthesized CDs and prepared nanocomposite films were analyzed using Fourier transform infrared (Jasco FT/IR-6700) spectroscopy. All spectra were measured in the wavenumber range from 4000 to 500 cm^–1^. X-ray photoelectron spectroscopy (XPS) analysis of CDs was performed on a Specs-Flex XPS system with Al/Kα (1486.7 eV) as the source. X-ray diffraction (XRD) analyses of the multicolor CDs and CD/PVA films were carried out by using a Rigaku Smartlab Intelligent X-ray diffractometer (Rigaku Americas, Texas USA). The samples were recorded at 2θ values between 5 and 80° and a scan speed of 5° min^–1^ was applied. Thermogravimetric analysis (TGA) of CDs and the nanocomposite films were determined using a TGA instrument, Mettler Toledo-TGA 3+. The samples were heated at a rate of 10 K/min from 25 to 800 °C under a nitrogen atmosphere at a flow rate of 40 mL/min. Absorbance values for the cytotoxicity study were measured using a microplate reader (Synergy HTX-BioTek, Winooski, VT, USA).

### 2.3 Synthesis of Carbon Dots

#### 2.3.1 Synthesis of Blue Carbon Dots

Blue carbon dots (BCDs) were synthesized according to our previously reported method.^55^ In the BCD synthesis, carob molasses, polyethylene glycol (PEG), and urea were used as the carbon source, passivation agent, and nitrogen (N) doping agent, respectively. To prepare nitrogen-doped BCD, 1 g of carob molasses was diluted in a 1:10 ratio in deionized water and 1 mL of this solution was mixed with the passivating and N doping agents (1:8:4 w/w) dispersed in 2 mL of (1:1 v/v) water/ethanol. Then, the obtained mixture was heated at 250 °C for 45 min in a Teflon oven vessel. The resulting material was dissolved in 6 mL of deionized water and this suspension was centrifuged at 13,500 rpm for 20 min to remove large particles. The supernatant was taken and vacuum-dried at 90 °C overnight.

#### 2.3.2 Synthesis of Green Carbon Dots

Green carbon dots (GCDs) were synthesized using the same method by changing the carbon source to lemon salt. To do so, 0.12 g of lemon salt was mixed with 0.12 g urea (2 mmol, N-doping agent) and dissolved in 8 mL of water/ethanol (1:1 v/v).^61^ This obtained suspension was transferred to a Teflon oven vessel and heated at 250 °C for 45 min until carbonization occurred. The resulting material was dissolved in 6 mL of Milli-Q water. Subsequently, the dark brown solution was centrifuged at 13500 rpm for 20 min to remove large particles and the supernatant was collected. The final product was vacuum-dried at 90 °C overnight.

#### 2.3.3 Synthesis of Yellow Carbon Dots

The yellow carbon dots (YCDs) were prepared with a modified version of the method described in a previous report.^55,62^ Lemon salt (1 g) and urea (2 g) were dissolved in 10 mL of DMF and obtained transparent solution was transferred into a Teflon oven vessel. Then, this solution was heated at 180 °C in the oven for 80 min and cooled down to room temperature. The obtained dark brown solution was centrifuged (13,500 r min^–1^, 20 min), and the surfactant was taken and vacuum-dried at 90 °C overnight.

#### 2.3.4 Synthesis of Red Carbon Dots

Red carbon dots (RCDs) were synthesized using the same method as previously reported.^63^ First of all, 0.028 g of paraphenylenediamine was sonicated in 25 mL of ethanol/water (1:1, v/v) until a homogeneous solution was obtained. The obtained mixture was taken into Teflon containers and tightly closed. The reaction was carried out in a microwave synthesis device at a constant temperature of 180 °C for 1 h. The obtained solution after the reaction was dried in an oven at 90 °C. The resulting RCDs were purified by silica column chromatography using mixtures of DCM and MeOH.

#### 2.3.5 Preparation of Multicolor Photoluminescent PVA and CD/PVA Nanocomposite Films

**Scheme 1:**
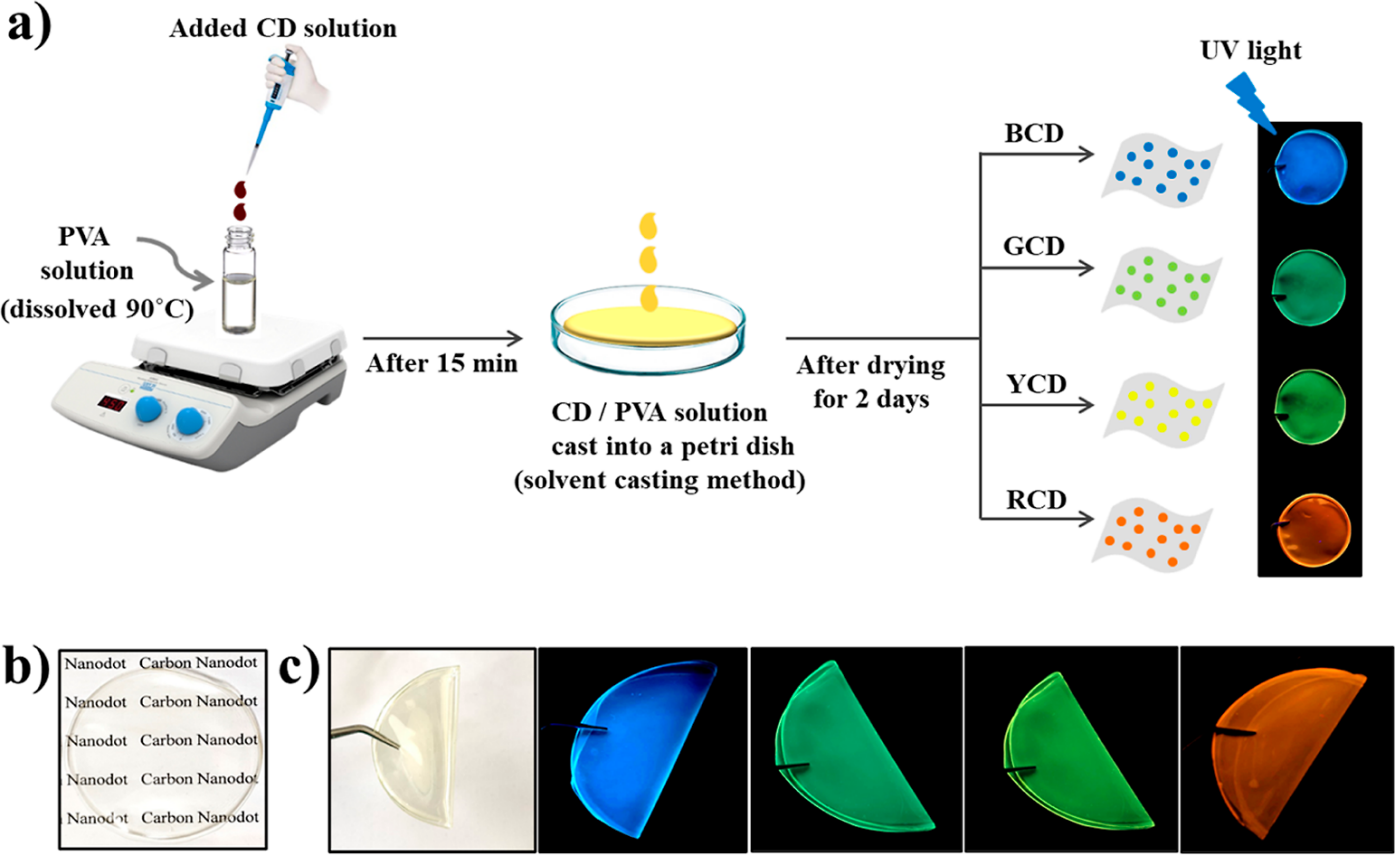
(a) Illustration of the preparation procedure for multicolor photoluminescent CD/PVA nanocomposite films. Digital images of the films present their (b) transparency and (c) flexibility with emission colors exhibited under UV light.

Multicolor CD/PVA nanocomposite films were produced by loading changing concentrations (0.25 to 2 wt.%) of CDs that exhibit different emissions to the PVA suspension. First of all, 1.5 g of PVA was dispersed in 19 mL deionized water and heated up to 95 °C under vigorous stirring using a magnetic stirrer for about 30 min until a transparent solution was obtained. Afterward, the produced solution was cooled down to room temperature (RT, 25 °C). Then, 1 mL of CD solutions at a certain mass concentration (0, 0.25, 0.5, 1.0, and 2.0 mg/mL) are added to the PVA solution. The mixture was then subjected to a combination of stirring/sonication cycles for 15 min until a homogeneous solution (Figure S1) was obtained. Thereafter, 4 mL of obtained PVA or CD/PVA solutions were cast into a glass petri dish using a micropipette and dried at room temperature for 2 days to allow the film to be formed. The film formation takes place with controlled evaporation of water. The same procedure was followed in the preparation of all CD/PVA nanocomposite film samples. The film thickness was controlled during the preparation by using the same amount of CD/PVA solution and keeping the film sizes as stable as likely (Scheme 1a). The thickness of the prepared casted films was measured to be about 0.11 ± 3 mm by LCD digital vernier calipers. Sample names were abbreviated as PVA, 0.015CD/PVA, 0.03CD/PVA, 0.05CD/PVA, and 0.10CD/PVA considering the percentage weight composition of CD/PVA films, which were tabulated in Table S1**.** Samples were preserved and monitored for over a year in a stability chamber at 25 °C (dark), 75% RH, and optical properties and film structure were monitored for over a year.

#### 2.3.6 Determination of In Vitro Cytotoxicity of CD/PVA Nanocomposite Films by the MTT Assay

Cytotoxicity of PVA and CD/PVA nanocomposite films on MRC-5 (human lung fibroblast healthy cell line) was determined using the MTT cell viability method. MRC-5 cells (1 × 10^4^ cells/well) were seeded to 96-a well plate in MEM supplemented with 10% FBS and 1% l-glutamine and incubated at 37 °C in a 5% CO_2_ incubator. 24 h later, PVA, BCD/PVA, GCD/PVA, YCD/PVA, and RCD/PVA in liquid form were applied to the fibroblast cells at a total concentration of 25, 50, 100, 250, and 500 mg/mL. After 24 and 48 h later, the medium was changed with 10% MTT solution prepared in a fresh medium, and plates were incubated in dark for 3 h. After the incubation period, MTT solutions in each well were discarded and 100 μL of DMSO was added to each well to dissolve the formazan crystals which were formed according to the mitochondrial activity of cells, and plates were shaken for 15 min. Then, the absorbance of each well was measured at 570 nm. 500 μM (or 17 mg/L) H_2_O_2_ was used as the positive control (PC) while PBS was used as the negative control (NC).

Percentage cell viability (%) was calculated as follows
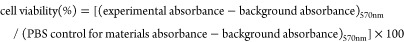
1

The results were shown as the mean and standard deviation (mean ± standard deviation) of at least three replicates. For cell viability experiments, data were analyzed for statistical significance using one-way ANOVA. An unpaired *t*-test with Welch’s correction was used for further analysis to compare differences between each sample group. GraphPad Prism 8.4.3 was used for all statistical analyses. Significant differences among sample groups were indicated as *p* < 0.05(*), *p* < 0.01(**), and *p* < 0.0001(****).

#### 2.3.7 DPPH Scavenging Activity of CD/PVA Nanocomposite Films

The antioxidant activities of CD/PVA nanocomposite films were experimented with by the DPPH free radical scavenging process.^64^ A 2.0 mL of 0.002% DPPH was added to 500 μL of various levels (25–500 mg/L) of CD/PVA films. Afterward, they were incubated for half an hour at 25 °C in the dark and the antioxidant activities were measured at 517 nm using a UV–vis spectrophotometer. Trolox and ascorbic acid were used as the standard. The activity to scavenge DPPH free radicals were calculated by the following equation
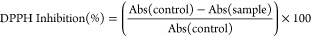
2where Abs_control_ is the control absorbance and Abs_sample_ is the CD/PVA films or standard absorbance after 30 min.

#### 2.3.8 Ferrous-Ion Chelating Activity of CD/PVA Nanocomposite Films

The chelating abilities of CD/PVA nanocomposite films for Fe^2+^ were tested using the procedure by Dinis et al..^65^ First, 0.5 mL of CD/PVA film solution with different concentrations ranging from 25 to 500 mg/L was added to a solution of FeCl_2_. The reaction was initiated by the addition of ferrozine and incubated at 25 °C for 10 min. The absorbance was spectrophotometrically determined at 562 nm. EDTA solution was utilized as a positive control. The chelating ability of the CD/PVA films for Fe^2+^ was calculated using following eq 1
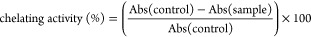
3where Abs_control_ is the absorbance of the control reaction and Abs_sample_ represents the absorbance obtained in the presence of CD/PVA films or EDTA.

#### 2.3.9 Biofilm Inhibition Activity of CD/PVA Nanocomposite Films

The biofilm inhibition of CD/PVA nanocomposite films was performed by using *Staphylococcus aureus*. Various concentrations of CD/PVA films were added to the wells and then wells were inoculated with *S. aureus*. The plates were incubated at 37° for 72 h. After 72 h, the medium was poured out from the plates and the wells were rinsed two times with distilled water. The plates were kept dry at 80° for 30 min and then crystal violet was added to each well and held for 60 min. After 60 min, the crystal violet was poured out from the wells and each well was cleaned two times with distilled water. Then, alcohol was added to each well and the measurement was taken on a spectrophotometer at 595 nm. The wells with no compound were used as a control. The biofilm inhibition of CD/PVA films was calculated according to the formula below.
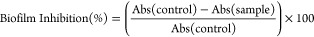
4where Abs_control_ is the control absorbance and Abs_sample_ is the CD/PVA film absorbance.

#### 2.3.10 DNA Cleavage Ability of CD/PVA Nanocomposite Films

The DNA cleavage abilities of CD/PVA nanocomposite films were investigated by agarose gel electrophoresis using pBR322 plasmid DNA. DNA molecules and CD/PVA films were incubated at 37 °C for 45 min at 250 and 500 μg/mL. After that loading dye was added and these solutions were electrophoresed for 1.5 h at 50 V by using 1% agarose gel. The agarose gel was visualized and photographed under UV light.

#### 2.3.11 Antimicrobial Activity of CD/PVA Nanocomposite Films

Antibacterial activities of CD/PVA nanocomposite films were performed by a broth microdilution procedure. *Bacillus cereus*, *Escherichia coli* (ATCC 10536), *S. aureus* (ATCC 6538), *Legionella pneumophila* subsp. *Pneumophila* (ATCC 33152), *Enterococcus hirae* (ATCC 10541), *Pseudomonas aeruginosa* (ATCC 9027), and *Candida albicans* were used as test microorganisms for assessing antimicrobial activities of CD/PVA films*.* The cultures were grown overnight prior to the testing. The microorganism growth media were inoculated with 3.2 × 10^8^ cfu/mL. Twofold serial dilutions of CD/PVA films were prepared in 96 well plates. The plates were incubated at 37 °C for 24 h. After that, minimum inhibition concentration (MIC) values were determined as the lowest concentration of CD/PVA films that could inhibit the growth of the microorganisms.

#### 2.3.12 Coating of Strawberries with PVA and CD/PVA Nanocomposites

Fresh strawberries were supplied from the grocery. These strawberries were attentively chosen to be uniform in terms of size and shape, color, texture, and visible defects, blemishes, and decay before coatings were applied. First, the strawberries are washed and dried. The cleaned strawberries were coated two times in PVA, 0.05GCD/PVA, and 0.05RCD/PVA solution by the dip-coating method. Then, the uncoated and coated strawberries were hung on the apparatus prepared for drying at room (25 °C) and fridge (4 °C) temperature. The images of the strawberries were taken using a smartphone between 0-12 days for visual evaluation. In addition, half of 1 strawberry was coated with 0.05GCD/PVA and examined by means of mold growth (see Video S5 and Figure 6a). Repetitions were made with at least four strawberries.

## 3 Results and Discussion

**Figure 1:**
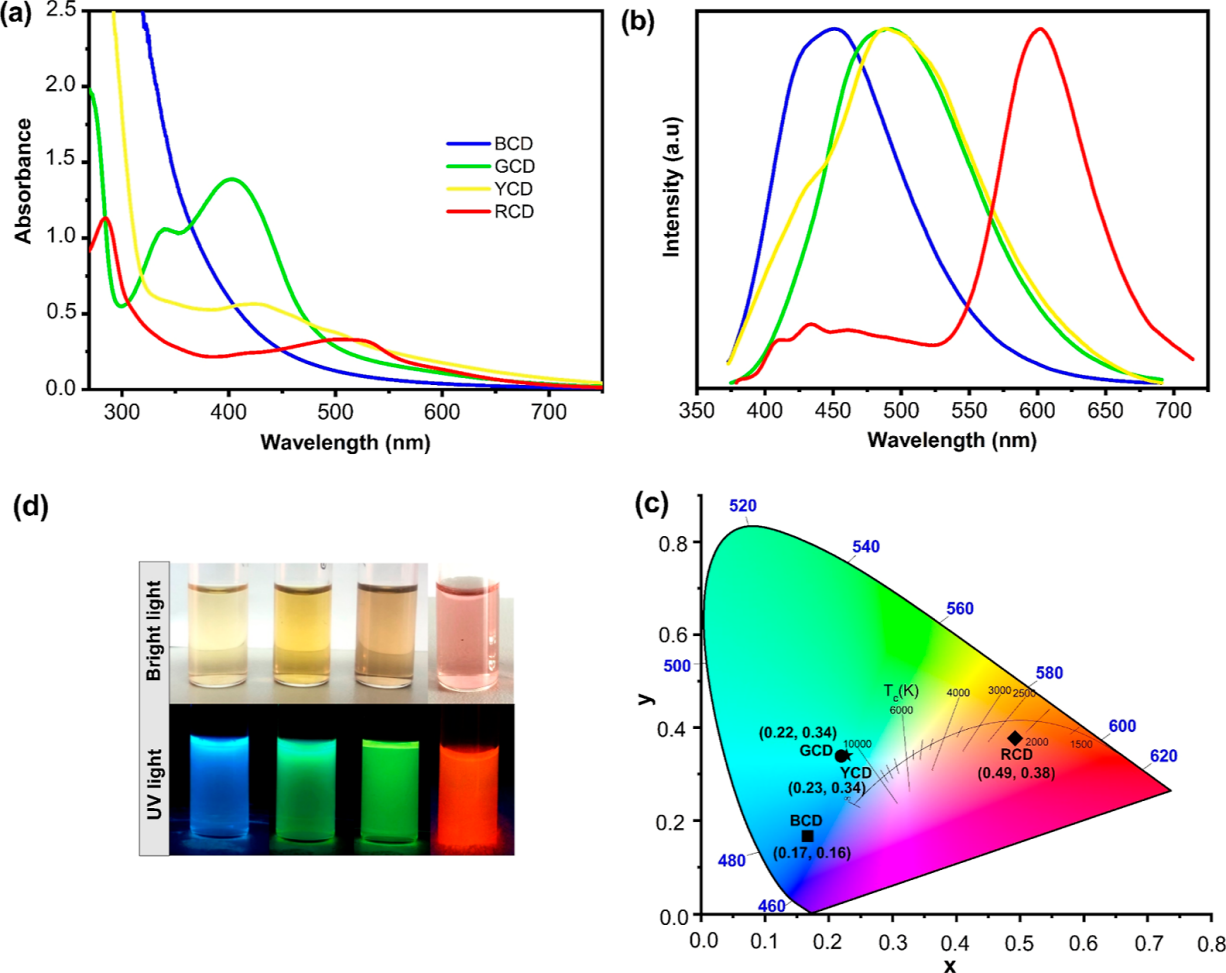
(a) UV–vis absorption spectrum, (b) normalized fluorescence emission at 365 nm excitation wavelength spectrum of multicolor CDs, (c) images of CDs under bright and UV light, and (d) calculated CIE coordinates from the PL spectra of CDs in solution at an excitation source (365 nm).

The image in Scheme 1b shows the optical transparency of the resultant nanocomposite film overlaying a black text logo. Moreover, these nanocomposite films have a flexible structure as well as high transparency under sunlight (see Videos S1, S2, S3, and S4, and Scheme 1c). Figure 1a shows the UV–vis spectrum of synthesized CDs. As seen in the graph, BCD showed apparent optical absorption in the UV region with a tail extending to the visible range (blue).^66−68^ The optical absorption peak of the GCD was observed in the UV region with a maximum absorption peak at about 340 nm and 405 nm. This is attributed to the n−π transition of C=O or C=N bonds, which defines the presence of aromatic structures with the surface or molecular center of the particles.^61,69^ For the YCD, the absorption peaks at around 350 nm and 430 nm were appointed to the absorption of C=O and C=N groups on the surface of CD.^70^ RCD exhibits UV/vis absorption from 250 nm to 510 nm. Specific peaks in the 255 and 285 nm are ascribed to the π–π* transition of electrons in aromatic moieties. Additional absorption peaks centered at 510 nm can be attributed to the n−π* transition C=O and C=N bonds, defining the presence of aromatic structures.^70−72^

The fluorescence center of CDs is associated with carbon-core states, surface defect states, and surface functional groups. Fluorescence in CDs is attributed to intrinsic and extrinsic fluorescence associated with the localized sp^2^ carbon field and surface states. In carbon-core states, the fluorescence center depends on the band gap of the conjugated π-domains. The larger the size of the CDs with the conjugated π-domain, the smaller the band gap (Figure S6a).^73,74^Figure S6b–e shows the band gap values of CDs, which were calculated using the Tauc-plot method. The band gap energies of BCD, GCD, YCD, and RCD were calculated to be 3.0, 2.65, 2.32, and 2.15 eV, respectively. As the size of CDs increased, the band gap caused by π-electron delocalization in the sp^2^ domain gradually reduced, which in turn led to the emission wavelength being red-shifted from blue to red.^75^

The synthesized CDs exhibited bright blue, green, yellow, and red fluorescence colors when irradiated with a UV light (λ = 365 nm) source with maximum emission peaks centered at 451, 490, 492, and 601 nm, respectively (Figure 1b,c). The quantum yield of the BCD, GCD, YCD, and RCD at an excitation wavelength of 365 nm was calculated to be 24.4, 32.3, 13.7, and 29.2%, respectively (see Figures S2–S5 for measurement details). Additionally, the Commission Internationale de L’’Eclairage (CIE) chromaticity values were calculated from the emission spectra of CDs, and they are depicted in Figure 1d. The CIE coordinates were located at (0.17, 0.16), (0.22, 0.34), (0.23, 0.34), and (0.49, 0.38) for blue-green, yellow, and red CDs, respectively, which is compatible with the PL colors of CDs in solution.

^1^H NMR spectra of CDs are represented in Figure S7 revealing the existence of different chemical environments in different regions which also varied with the type of the CDs. The regions identified in the ^1^H NMR spectra for multicolor CDs in Figure S7 are as follows: in the ^1^H NMR spectrum of BCDs, the regions found were 0.5–1.3 ppm (for sp^3^ C–H protons), 3.0–3.3 ppm (for alcohol −OH groups), and 3.3–5.0 ppm (for protons bound by hydroxyl, ether, and carbonyl groups). In the ^1^H NMR spectrum of GCDs, the regions found were 1.0–1.3 ppm (for sp^3^-hybridized C–H protons), 1.3–2.3 ppm [for amine −NH protons)] 3.3–5.0 ppm (for protons bound by hydroxyl, ether, and carbonyl groups), and 5.0–7.0 ppm (for alkene −CH or amide N–H protons). The ^1^H NMR spectrum of YCDs differed from GCDs as the regions between 3.3 and 8.0 ppm did not appear in the YCD sample. In the ^1^H NMR spectrum of RCDs, the regions found were 0.5–1.3 ppm (for sp^3^ C–H protons), 2.3–3.0 ppm (for amine N–C–H protons), 5.0–7.0 ppm (for alkene −CH or amide N–H protons), and 6.0–8.0 ppm (for aromatic C–H protons).^76^

**Figure 2:**
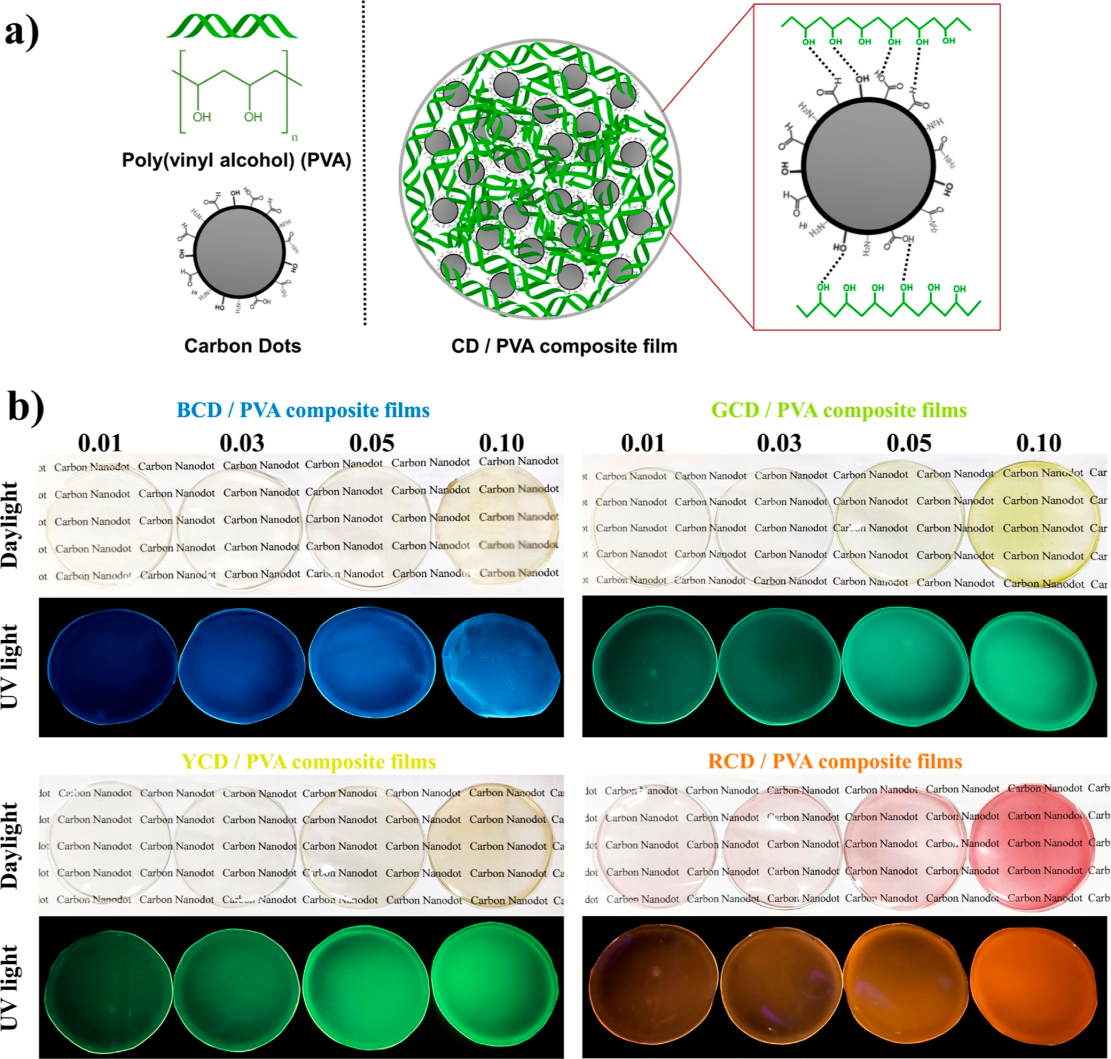
(a) Scheme representing the hydrogen bonding interaction between the PVA matrix and CDs’ role in the nanocomposite film formation and (b) digital images of multicolor photoluminescent CD/PVA nanocomposite films consist of varying concentrations of CDs (0.01–0.1 w/w % in CD/PVA mixture) under daylight (up) and 365 nm UV light (down).

Figure 2a represents the formation mechanism of the nanocomposite films by the formation of hydrogen bonding between the hydroxyl (-OH) groups of PVA and oxygen-containing functional groups of CDs. All of the CD/PVA nanocomposite films showed good transparency under daylight even at the highest CD concentrations and exhibited high-intensity emission colors similar to the CDs they contain when stimulated under 365 nm UV light, while the PL intensity of the films increased as the CD concentrations increased (Figure 2b).

**Figure 3:**
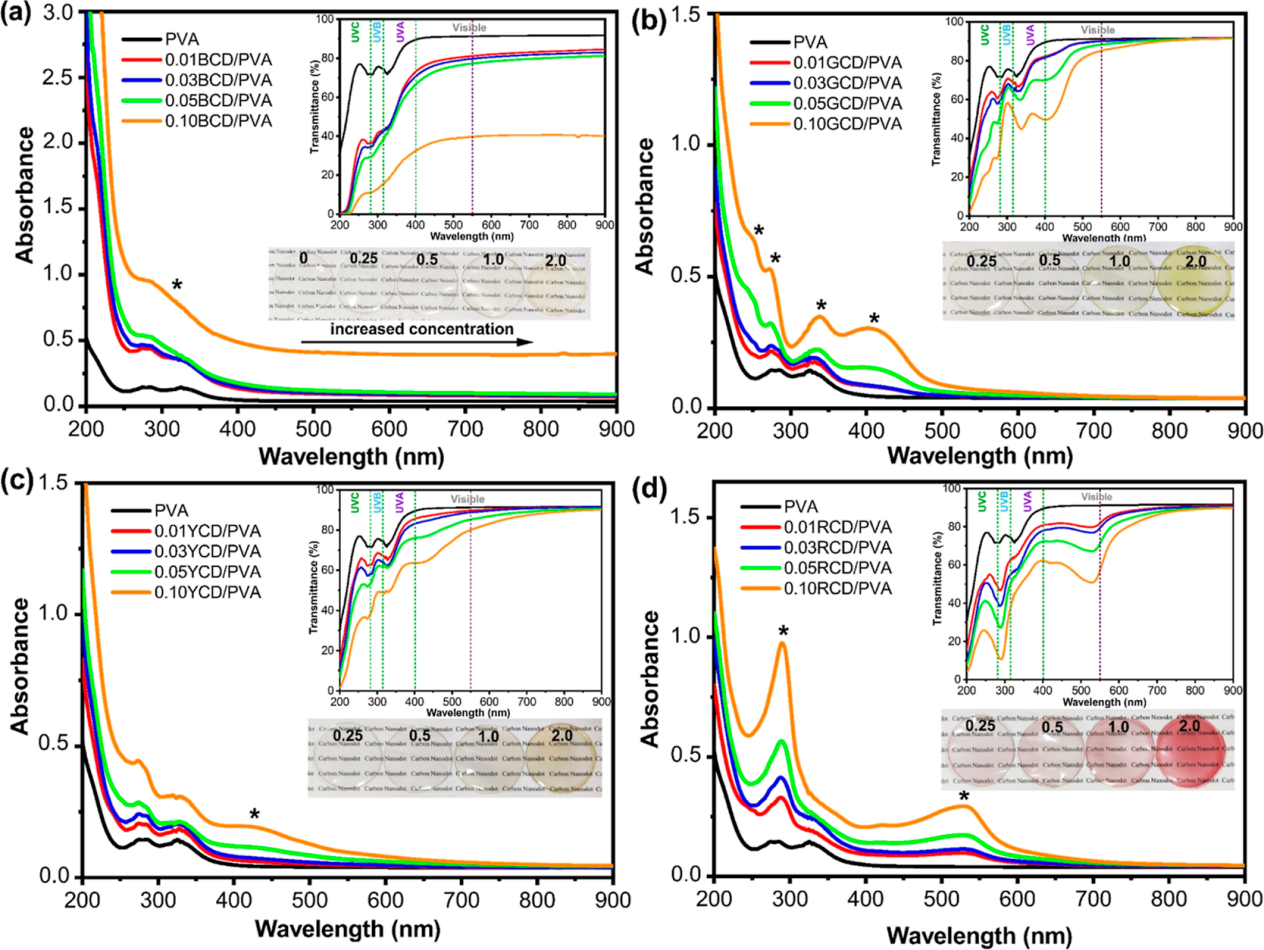
UV–vis absorption spectrum of (a) BCD/PVA, (b) GCD/PVA, (c) YCD/PVA, and (d) RCD/PVA nanocomposite films prepared at varying CD concentrations (inset: transmittance spectra and photographs under daylight of (a) BCD/PVA, (b) GCD/PVA, (c) YCD/PVA, and (d) RCD/PVA nanocomposite films).

The optical properties of PVA and CD/PVA nanocomposite films were characterized by UV–vis spectroscopy, as shown in Figure 3. PVA film alone transmitted 91.2% of the light in the visible region (400–800 nm). After the integration of different concentrations of CDs into PVA, a gradual decrease in the transmittance of visible light was observed. The optical transmittance for BCD/PVA, GCD/PVA, YCD/PVA, and RCD/PVA nanocomposite films at 550 nm at the highest CD content (0.10 CD/PVA) was measured to be 39.68, 85.16, 80.16, and 81.96%, respectively (Figure 3, Table 1). The slight decrease in the optical transparency of CD/PVA nanocomposite films might be due to the good dispersion of multicolored CDs through the PVA matrix, which has little effect on the transmittance of nanocomposites. The peaks observed in the absorption spectrum of CD/PVA films, unlike the PVA film, belong to the characteristic peaks of CDs (Figure 3) and are evidence of the doping of CDs into the PVA polymer matrix (the characteristic peaks of the CDs are indicated by a black asterisk). Moreover, it was observed that the pure PVA film showed low absorbance in the UV region from 200 to 400 nm, and the inclusion of multicolored CDs in the PVA film increased the light absorption in the UV region even at the lowest CD concentrations (0.01 CD/PVA).

**Table 1:** Transmittance Values of Pure PVA and CD/PVA Nanocomposite Films in UV-A, UV-B, and UV-C Regions

transmittance (%)				
samples	UVC (250 nm)	UVB (300 nm)	UVA (350 nm)	visible (550 nm)
PVA	**76.86**	**75.68**	**79.25**	**91.24**
0.01BCD/PVA	35.17	41.54	54.27	81.12
0.03BCD/PVA	28.76	39.62	55.32	79.69
0.05BCD/PVA	21.62	34.24	51.48	77.25
0.10BCD/PVA	**7.78**	**12.96**	**23.23**	**39.68**
0.015GCD/PVA	62.42	70.71	72.49	90.16
0.03GCD/PVA	55.95	67.75	70.56	90.02
0.05GCD/PVA	35.31	66.10	64.32	88.32
0.10GCD/PVA	**22.78**	**58.15**	**48.59**	**85.16**
0.01YCD/PVA	63.33	68.79	72.62	89.75
0.03YCD/PVA	60.22	65.32	70.04	88.81
0.05YCD/PVA	49.93	61.75	67.31	85.26
0.10YCD/PVA	**33.94**	**48.82**	**55.13**	**80.16**
0.01RCD/PVA	52.72	54.24	70.80	81.96
0.03RCD/PVA	50.51	46.16	64.35	79.46
0.05RCD/PVA	41.01	35.41	61.12	71.31
0.10RCD/PVA	**25.54**	**17.68**	**51.67**	**57.27**

Following these results, we further investigated the transmittance of PVA and CD/PVA nanocomposite films at particular wavelengths corresponding to different UV light rays (UVA, UVB, and UVC). As seen in Table 1, films produced by CD integration to the PVA transmitted less amount of UV light as compared to the pristine PVA films in which the ratio depended on the CDs’ PL emission color. The 0.10BCD/PVA, 0.10GCD/PVA, 0.10YCD/PVA, and 0.10RCD/PVA nanocomposite films with the highest CD content adsorbed the light by 92.22, 77.22, 66.06, and 74.46% at the UVC region, 87.04, 41.85, 51.18, and 82.32% at the UVB region, and 76.77, 51.41, 44.87, and 48.33% at the UVA region, respectively, while the PVA film alone transmitted 23.14% at UVC, 24.32% at UVB, and at 20.75% UVA region. BCD-incorporated PVA films showed the highest UV-shielding efficacy out of the four CDs studied. Although increased CD content in the PVA film matrix led to improved shielding efficacy for UVC, UVB, and UVA regions, it also resulted in a slightly reduced transparency (Figure 3). All these results showed that the addition of multicolor CDs to the PVA film can be used as an effective UV absorber in the production of transparent UV-shielding films without affecting their transparency under visible light. Moreover, the emission spectra recorded for the CD/PVA nanocomposite films (Figure S8) showed similar characteristics to the emission spectra of CDs in solution. This confirmed that the polymer matrix did not disturb the photophysical properties of the embedded CDs.

Figure S9 shows TEM images of CDs and corresponding FE-SEM images of multicolor 0.05CD/PVA nanocomposite films. The mean particle size and standard deviation were calculated by measuring the diameter of at least 100 particles using ImageJ. TEM images display that they are homogeneous and well dispersed with the particle size distributions of 11.61 ± 2.75, 19.78 ± 3.52, 24.48 ± 5.12, and 38.72 ± 8.21 nm for BCD, GCD, YCD, and RCD, respectively (Figure S8b–e). As indicated previously in the optical characterization section, TEM imaging also demonstrated the effect of the increased particle size on their red-shifted fluorescence emission maxima in solution. SEM images represented in Figure S9a, the PVA film displayed a smooth surface while the accumulated BCDs were visible on the 0.05BCD/PVA nanocomposite film surface. In the case of films consisting of GCDs, YCDs, and RCDs, a good distribution of the CDs through the PVA matrix was observed (Figure S9c–e).

CDs’ elemental composition and dominating surface chemical bonds were determined by the XPS technique. As can be seen from the survey scan given in Figure S10, three distinct peaks at about 287, 401, and 533 eV were appointed to the 1s orbital of the carbon atoms, nitrogen, and oxygen, respectively, confirming the presence of C, O, and N elements. Detailed functional groups for each CD type were characterized using high-resolution XPS (HR-XPS) spectra of C 1s, O 1s, and N 1s (Figure S11). In the C 1s HR-XPS spectra of the CDs, the C–C bond was observed at around 283.18–283.9 eV.^77^ In addition, peaks of the C–C/C=C (284.1–284.9 eV) bond were observed for BCD, GCD, and RCD, and peaks of the C=O (287.2–287.6 eV) bond were observed for BCD, GCD, and YCD.^78,79^ Moreover, YCDs had peaks of the C–O bond (285.2 eV) while YCD and RCD both presented peaks attributed to the C–N bond (286.3 eV).^80,81^ The high-resolution N 1s spectra in Figure S11 confirmed the presence of pyridinic N bonds (397.4–398.9 eV) in all CDs.^82^ In addition, peaks of the C–N–C (399.5–399.7 eV) bond were observed for GCDs and RCDs, whereas peaks of pyrrolic N (400.2–400.9 eV) bonds were observed only for GCD.^82,83^ High-resolution O 1s spectra show that all CDs have peaks of C=O (529.5–530.9 eV) bonds.^84−86^ Besides, peaks attributed to C–OH/C–O–C (531.2–533.1 eV) bonds were observed in all CDs except YCDs.^86,87^ Variation of the surface functional groups not only affects the defect states, and thus, the optical properties of the CDs, but also we expect that it would also further be effective on the biological activities of the CDs which will be discussed in the next sections.

Figure S12 shows the FT–IR spectra of the powder CDs, PVA, and 0.05CD/PVA nanocomposite films. When the spectra of multicolored CDs were examined, all CDs showed absorption bands of OH/N–H stretching vibration between 3500 and 3100 cm^–1^. These functional groups increase the hydrophilicity and stability of fluorescent CNPs in aqueous systems. In addition, the peaks at approximately 1715–1700 cm^–1^ and 1600–1500 cm^–1^ in the FTIR spectra of CDs were attributed to the carboxylic O–C=O and aromatic C=C tension vibrations, respectively. Moreover, the vibrations at 1400–1300 cm^–1^, 1140–1050 cm^–1^, and 760 cm^–1^ were attributed to the sp^3^ C–H band, C–O–C band, and aromatic sp^2^ C–H (CH_2_) band, respectively. The characteristic stretch band of the C–NH–C bond of the amine groups on the surface of all CDs is observed at the peaks between 1200 and 1100 cm^–1^. These peaks are due to the amine functional groups formed on the CD surfaces due to nitrogen-containing precursors used during the synthesis. In addition, BCD and YCD exhibited peaks representing sp^3^ C–H stretching vibration between 3000 and 2800 cm^–1^. GCDs, on the other hand, exhibited peaks of aldehyde C–H stretching vibration between 2700 and 2760 cm^–1^. Unlike other CDs, RCD showed the N–H band and vibration band of out-of-plane deformation of 1,4-disubstituted benzene ring at 1512 and 825 cm^–1^, respectively, originating from paraphenylenediamine. These XPS and FT-IR results demonstrated that multicolor CDs are formed with a sp^2^-conjugated domain of aromatic structure, and the surface oxygen and nitrogen-including groups varied with respect to the fluorescence emission color of the CDs.

The incorporation of CDs into the PVA matrix was analyzed by analyzing the FT-IR spectrum of 0.05 CD/PVA composite films (Figure S12). The plane PVA film showed a broad peak between 3500 and 3000 cm^–1^ which was attributed to the −OH stretching vibration from intermolecular and intramolecular hydrogen bonds of PVA. Also, the bands stretching of the C–H alkyl groups were observed at 2905 cm^–1^, the peak at 1659 cm^–1^ corresponds to C=O and C=C group in the PVA polymer matrix, while the band observed at 1080 cm^–1^ was belong to the hydroxyl C–O stretching. The films also showed peaks of CH/CH_2_ deformation vibration bands at 1300–1500 cm^–1^. Peaks attributed to C–C stretching and CH_2_ stretching vibrations were observed at 916 and 836 cm^–1^.^88^ Compared with the PVA film, the −OH stretching peak position slightly shifted in the CD/PVA nanocomposites suggesting the presence of hydrogen bonding interactions between the hydroxy groups of the PVA and hydroxyl groups of CDs’ surface. The inclusion of CDs into the PVA matrix did not result in any additional peak or change in the spectra which might be due to overlapping peaks and the low concentration of CDs in the mixture.^89,90^

Figure S13a shows the XRD spectra of multicolor-emitting CDs. The sharp peaks appeared at BCDs at approximately 19.17° (amorphous carbon structures), 23.56° (highly disordered carbon atoms and graphene structures), 26.27° [(002) plane of graphitic carbon], and 29.64° corresponding to amorphous carbon structures.^48,62,91−93^ The peaks between 20 and 30° represented the presence of graphene-like nanostructures. YCDs and GCDs had single peaks at 27.3° corresponding to a highly disordered amorphous carbon structure.^93^ While the peaks centered at approximately 15.9 and 30.5° indicate the presence of amorphous carbon structures presented in RCDs, the broad peak centered at about 21° degrees represents the lattice spacing of graphite (002).^94−97^ We further investigated the interaction of CDs with PVA in nanocomposite films, and the results are depicted in Figure S13b. PVA polymer showed two typical peak diffractions. The first peak with high intensity appeared at 2θ = 19.36° and is attributed to the spacing of (101), which reflects the semi-crystalline properties of PVA. The second peak with low intensity at 2θ = 44.4° is ascribed to the semicrystalline nature of PVA. Compared to pure PVA, the XRD pattern of CD/PVA composite films did not lead to new crystal diffraction peaks but only resulted in a slight shift in the peak centers, which was expected considering that CDs were incorporated in the polymeric matrix at trace amounts (Table S1).^98,99^

The thermal stability of polymers and composites plays an important role in food packaging applications and can be enhanced by the incorporation of nanoparticles. TGA analysis was used to investigate the effect of the CDs on the thermal stability of CD/PVA films. Figure S14 shows the TGA thermograph of pristine PVA film and CD-integrated PVA of varying CD concentrations (0.25–2 mg/20 mL of CD/PVA mixture). TGA curves of PVA film and nanocomposite films showed three main steps of mass loss. The first step (I) showed the dehydration of the surface adsorbed water at 50–270 °C. The mass loss in the second step at 270–450 °C was related to the decomposition and carbonization of the PVA polymer.^100,101^ The *T*_50_ value, which is defined as the temperature at which 50% of the initial weight remains was used for determining the thermal stability of the tested material. As shown in Table S2, *T*_50_ values of nanocomposite films increased with the addition of CDs, indicating that all CD/PVA nanocomposite films are thermally more stable than pure PVA films at 50% weight loss. The T_50_ values were increased by a maximum of 12.9, 19.5, 24.8, and 22.3 °C with the addition of BCD, GCD, YCD, and RCD compared to the PVA film, respectively. The weight loss that occurred in the last step (from 450 to 800 °C) indicates that the remaining adsorbed polymer and CDs were completely decomposed. The addition of CDs to all films increased the amount of residue formed in the process of PVA decomposition. While the PVA alone decomposes entirely before 800 °C, up to 4.0% of black carbonized residues remain after the burnout of nanocomposite films (Table S2). The oxidative decomposition of graphite begins at 600 °C.^102^ The residue at 800 °C (up to 4.0%) might represent the percentage amount of CDs incorporated in the polymeric support (Table S2). According to the TGA curves, it is clear that the temperatures at which weight loss occurs and the amount of solid residues increased with the incorporation of CDs, indicating that CDs improve the thermal stability of CD/PVA nanocomposite films at elevated temperatures, which could be useful for many applications focused on protective coatings and films. That improvement in the thermal stability can be attributed to carbon back bounds of CDs homogenously distributed in the PVA, elimination of side hydroxyl groups of PVA, and strong interfacial interactions that inhibit oxidation, possibly due to the contact between the polymer and CDs.^103,104^ The prepared films were further monitored at ambient conditions (25 °C, dark, 75% RH) for over 1 year. We observed that they maintained their stability over time with no apparent change in their optical properties as well as their form.

**Figure 4:**
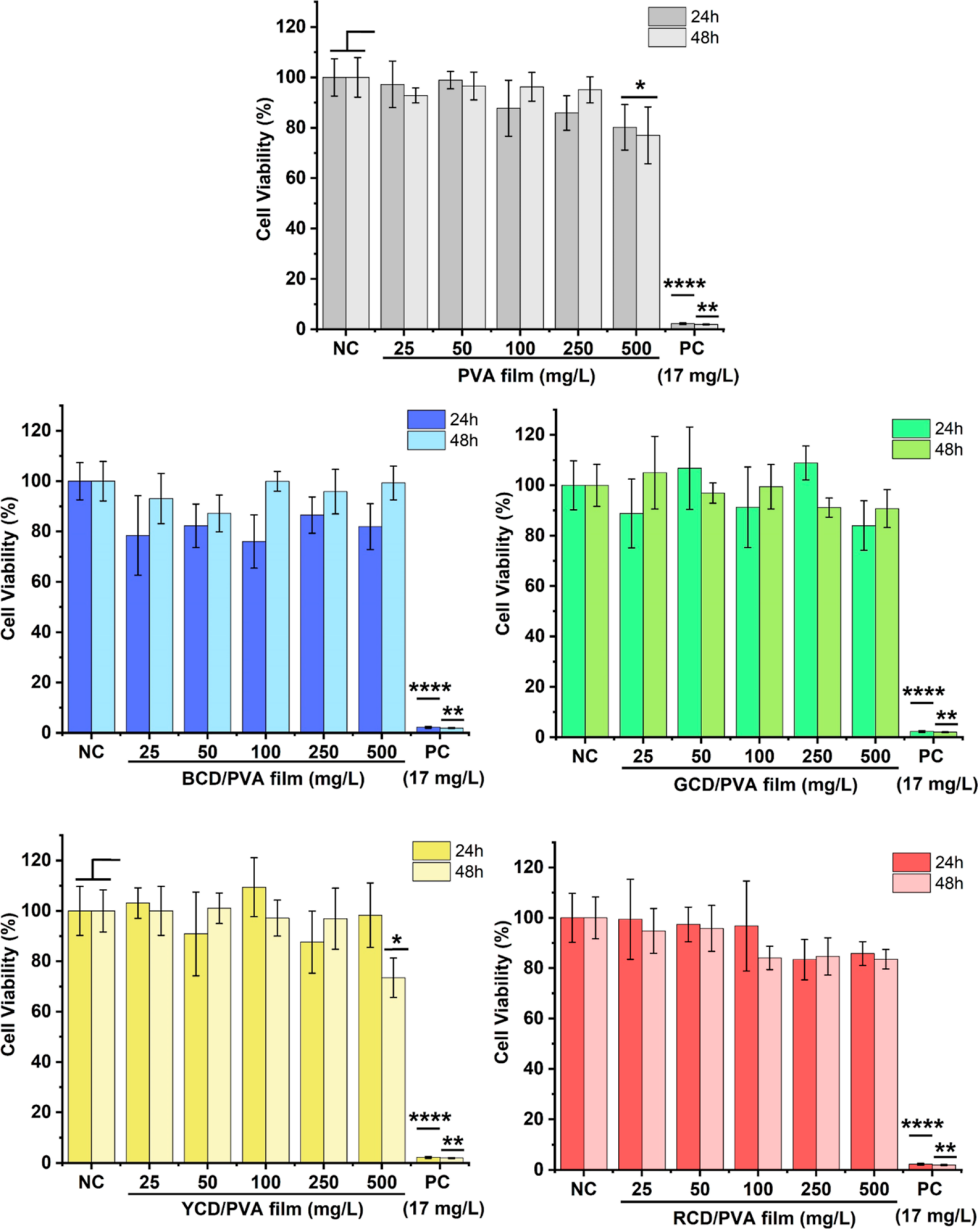
Concentration and time depended on cell viability of PVA and 0.05CD/PVA-treated MRC57 cells. The negative control (NC) was a PBS-containing medium and the positive control (PC) was H_2_O_2_, 17 mg/L. Values represent as mean ± SD with *n* = 4. Significant differences among sample groups were indicated as *p* < 0.05(*), *p* < 0.01(**), and *p* < 0.0001(****).

The MTT assay was used to evaluate the in vitro cytotoxicity of the PVA and 0.05CD/PVA films on the cell viability of MRC-5 (human lung fibroblast cell line) at the time (24 and 48 h)- and concentration (25, 50, 100, 250, and 500 mg/L)-dependent manners. Figure 4, shows the normalized cell viability of the MRC-5 treated with varying concentrations of the PVA film and multicolor emitting 0.05CD/PVA films (from 25 to 500 mg/L). The plane PVA films demonstrated a significant decrease in cell viability at the highest concentration (500 mg/L) after 24 h, while the 1CD/PVA samples were shown to be non-cytotoxic even at elevated composite concentrations. At 48 h, both PVA and 0.05YCD/PVA films at the highest concentration (500 mg/L) showed a significant decrease [*p* < 0.05(*)] in the cell viability, while the rest of the 0.05CD/PVA films (red, blue, and green) did not affect the cell viability at any concentration studied. Studies reported by Date et al. (2020) on cytotoxicity of CD-incorporated agarose PVA hydrogel nanocomposites on peripheral blood mononuclear cells (PBMC) were also consistent with our results where authors reported that CDs were not cytotoxic.^105^ These results demonstrated that these materials are non-toxic and can be potential candidates for food coating and packaging in the food industry.

**Figure 5:**
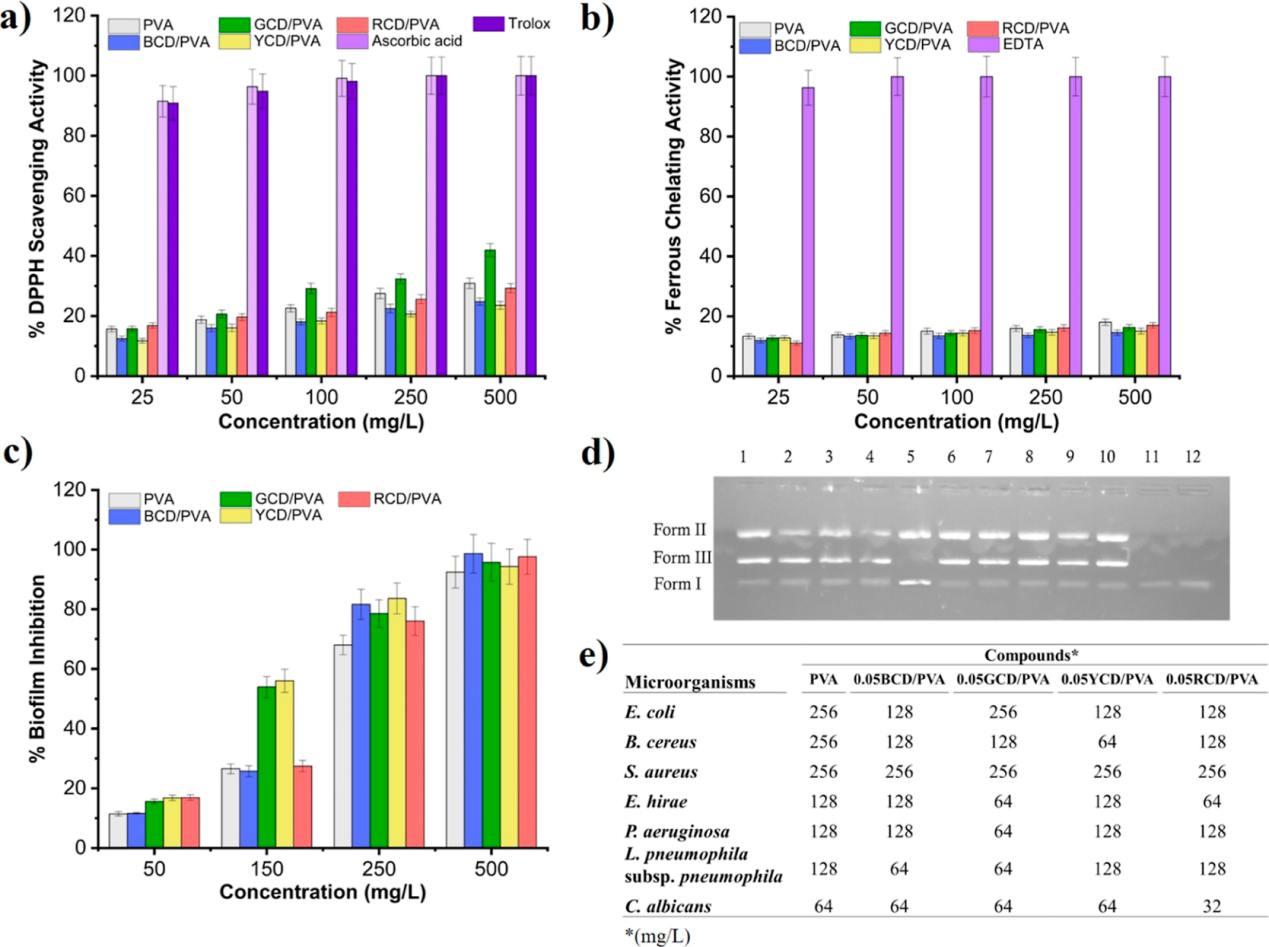
(a) DPPH scavenging activities, (b) metal chelating activities, (c) biofilm inhibition activities, (d) gel electrophoresis results of the DNA cleavage assay: lane (1) pBR 322 DNA + 250 μg/mL 0.05RCD/PVA, lane (2) pBR 322 DNA + 250 μg/mL of 0.05GCD/PVA, lane (3) pBR 322 DNA + 250 μg/mL of 0.05BCD/PVA, lane (4) pBR 322 DNA + 250 μg/mL of 0.05YCD/PVA, lane (5) pBR 322 DNA +250 μg/mL of PVA, lane (6) pBR 322 DNA + 500 μg/mL of 0.05RCD/PVA, lane (7) pBR 322 DNA + 500 μg/mL of 0.05GCD/PVA, lane (8) pBR 322 DNA + 500 μg/mL of 0.05BCD/PVA, lane (9) pBR 322 DNA + 500 μg/mL of 0.05YCD/PVA, lane (10) pBR 322 DNA + 500 μg/mL of PVA, lane (11) pBR 322 DNA, lane (12) pBR 322 DNA + DMSO, and (e) table of the MIC of tested microorganisms of PVA and 0.05CD/PVA nanocomposite films.

The antioxidant activities of nanocomposite films were evaluated by monitoring the DPPH scavenging activity and metal chelating activity. The DPPH solution color after using the PVA and 1CD/PVA nanocomposite film solution is discolored from violet to flavescent due to the free radical scavenging by antioxidants through the donation of H^+^ to create the stable DPPH–H molecule.^106^ As seen in Figure 5a, the antioxidant activities of PVA and 0.05CD/PVA films were slightly in a dose-dependent manner. When the concentration of 0.05BCD/PVA film increased from to 100 mg/L, the free radical scavenging activity increased from 12.48 to 18.05%. The free radical scavenging activities of PVA, 0.05BCD/PVA, 0.05GCD/PVA, 0.05YCD/PVA, and 0.05RCD/PVA nanocomposite films were found to be 27.49, 22.46, 32.35, 20.68, and 25.6%, respectively, at a concentration of 250 mg/L. The highest DPPH activity of 41.96% was obtained with the 0.05GCD/PVA film at a concentration of 500 mg/L. As FTIR and XPS results indicated that rich oxygen vacancies on the surface of CDs could play a role in the elevation of the adsorbed oxygen content and further might promote the activation of O^2^ resulting in ROS generation. This result is in line with the results of Kousheh et al (2020).^107^ Because excess free iron plays a role in the induction and generation of free radicals in biological organisms, it is particularly important to explore an antioxidant to remove free radicals. Therefore, we investigated the metal chelating capability of our newly synthesized 0.05CD/PVA films by using the metal chelating test. The result is represented in Figure 5b. Being tested in the concentration ranges of 25–500 mg/L, all test CDs demonstrated weak chelating activities. The metal chelating activities of PVA, 0.05BCD/PVA, 0.05GCD/PVA, 0.05YCD/PVA, and 0.05RCD/PVA films were 18.0, 14.51, 16.25, 15.03, and 16.95%, respectively, at 500 mg/L. The test compounds showed almost the same metal chelating activity at all tested concentrations.

A biofilm is a microbial community that is adhered to a surface and enclosed in extracellular polymeric materials. It is mostly composed of proteins, extracellular polysaccharides, nucleic acid lipids, and other chemical or biochemical substances. The microorganism in the biofilm can quickly permeate an extensive diversity of environmental spaces, including the human body.^108^ Moreover, biofilms are common in industrial, dental, medical, and marine environments, where they are undesirable because of their pathogenicity and tolerance to anti-biofouling technologies or antimicrobial drugs.^109^ For these reasons, inhibition of biofilms may play an important role in technological, ecological, and health-focused problems. *S. aureus*, selected due to its strong biofilm-forming activity supported in this investigation, was used in the assay of 0.05CD/PVA films. Figure 5c demonstrates the biofilm inhibition of PVA and 0.05CD/PVA nanocomposite films. The investigation of the effect of the CDs on the formed biofilm exhibited that the CDs were capable of inhibiting *S. aureus* biofilms. As seen in Figure 5c, the biofilm inhibition activity was found to be concentration dependent. When the concentration of PVA, 0.05BCD/PVA, 0.05GCD/PVA, 0.05YCD/PVA, and 0.05RCD/PVA films increased from 50 to 150 mg/L, the biofilm inhibition activities increased from 11.45 to 26.53%, from 11.64 to 25.73%, from 15.6 to 53.91%, from 16.8 to 56.0%, and from 16.91 to 27.43%, respectively. The biofilm inhibition activity at 250 mg/L of PVA, 0.05BCD/PVA, 0.05GCD/PVA, 0.05YCD/PVA, and 0.05RCD/PVA films were determined to be 68.0, 81.63, 78.57, 83.61, and 76.02%, respectively. The biofilm inhibition process displayed that the films also demonstrated biofilm inhibition activity in the order of PVA < 0.05YCD/PVA < 0.05GCD/PVA < 0.05RCD/PVA < 0.05BCD/PVA at a concentration of 500 mg/L. The highest biofilm inhibition was obtained with the 0.05BCD/PVA film at 98.6%. Similarly, Wang et al. reported the ability of graphene quantum dots to effectively eliminate *S. aureus* biofilms.^110^ The biofilm inhibition results revealed that the newly synthesized and characterized CDs demonstrated expressive biofilm inhibition activity. As a result, the tested CDs could be applied as biofilm inhibition agents after further pharmacological and toxicological tests.

Figure 5d shows gel electrophoresis of plasmid DNA with 250 and 500 mg/L concentrations of PVA and 0.05CD/PVA nanocomposite films after 90 min of incubation. When circular plasmid DNA is exposed to electrophoresis, the unbroken supercoil form (Form-I) relatively fast-moving migration will be seen. If cleavage takes place on one strand (nicking), the supercoil will relax to generate a slower migration open circular form (Form-II). If both strands are broken, a linear form (Form-III) that moves between Form-I and Form-II will be generated. As seen in Figure 5d, 0.05BCD/PVA, 0.05GCD/PVA, 0.05YCD/PVA, and 0.05RCD/PVA films exhibited double strain DNA cleavage activity at 250 mg/L concentration while only PVA demonstrated single strain DNA cleavage activity at the same concentration. According to previous investigations, the interaction between DNA and CDs was reported to lead to DNA secondary structural flexions by the terminal base pairs binding with oxygen-containing groups of CDs by electrostatic interactions and hydrogen bonding.^111,112^ Wang et al. investigated the interaction of graphene quantum dots with DNA molecules, and they also reported that GQDs caused DNA damage.^113^ These findings indicated that CDs could have the potential to be used as DNA targeting agents after being evaluated by toxicologic test systems.

The MIC values of PVA and 0.05CD/PVA nanocomposite films were studied by the double dilution process. The results are given in the table in Figure 5e. PVA, 0.05BCD/PVA, 0.05GCD/PVA, 0.05YCD/PVA, and 0.05RCD/PVA films were exhibited MIC values of 256, 128, 256, 128, and 128 mg/L against *E. coli*, respectively. Moreover, MICs of PVA, 0.05BCD/PVA, 0.05GCD/PVA, 0.05YCD/PVA, and 0.05RCD/PVA films against *B. cereus* were 256, 128, 128, 64, and 128 mg/L, respectively. Among the tested CDs, 0.05GCD/PVA was the most effective one against the test organisms. The most sensitive test microorganism was *C. Albicans* for all tested CDs. The highest antimicrobial activity was also determined with the 1RCD/PVA film as 32 mg/L against *C. Albicans*. Du et al. indicated that CDs effectively inhibited both Gram-negative (*E. coli*) and Gram-positive (*S. aureus* and *B. subtilis*) growth. They also reported that the MIC values of CDs against *E. coli* and *S. aureus* were found to be 192 and 384 μg/mL, respectively.^106^ Demirci et al. reported that CDs have inhibited the growth of both Gram-negative and Gram-positive bacteria,^114^ whereas our results showed that CD/PVA films displayed more effective antimicrobial activities than their findings. If bacteria infect human, they can lead to the generation of ROS, which is toxic to human health and influence DNA and may induce cancer. On the other hand, when CDs come into contact with bacteria in the presence of moisture, they generate highly reactive oxygen species, such as superoxide and hydroxyl radicals, and that can pass through the cell wall and lead to bacterial death.^115^ ROS can be presumably related to the direct intercalation into DNA, the deficiency of the bacterial DNA repair system.^116^ According to our DNA cleavage activity results, CDs also have good nuclease activities. As a result, the antimicrobial activity of the newly synthesized CDs against test organisms can be explained by all these mechanisms.

**Figure 6:**
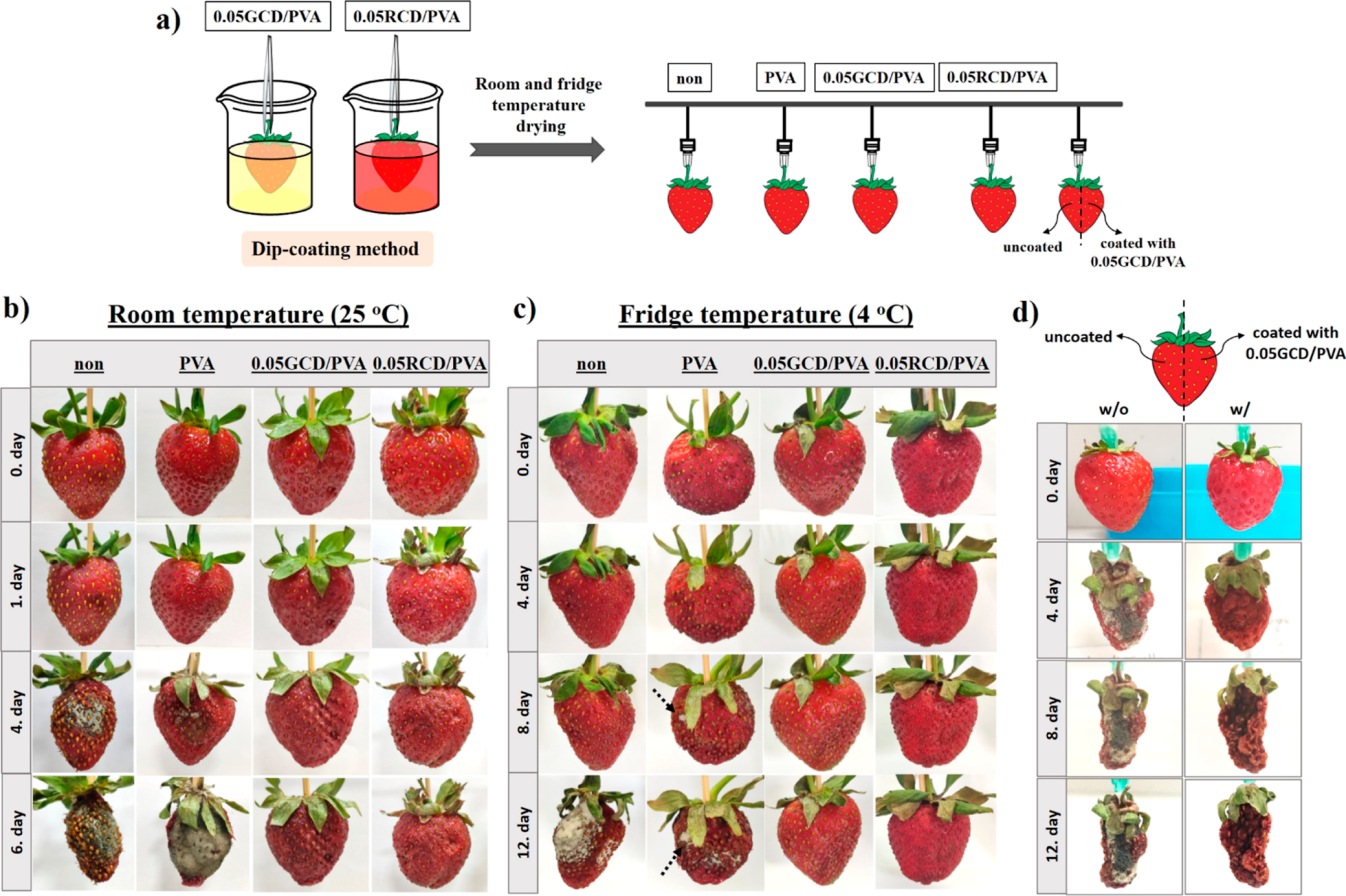
(a) Schematic illustration of strawberry coating with PVA and CD/PVA nanocomposites (green and red-emitting CDs) by the dip-coating method. Digital images of the appearance of non-coated and coated strawberries with PVA, 0.05GCD/PVA, and 0.05RCD/PVA at (b) room temperature, (c) fridge conditions, and (d) room-temperature images of 0.05GCD/PVA-coated and uncoated strawberries at varying storage times.

Taking into account the improved biological activities of CD-incorporated PVA films, we further set a visual demonstration for monitoring the mold formation on strawberries, which was chosen as a model for perishable fruits which need special conditions for long-term storage. Strawberries coated with PVA, 0.05GCD/PVA, and 0.05RCD/PVA (highest antimicrobial activity) were investigated at room and fridge temperatures. Coated and uncoated strawberries were photographed by a smartphone and changes in their appearances, mold formation, and water content were monitored visually (Figure S15). Compared to the strawberries kept at room temperature, no mold formation was observed at the end of the 6th day in CD/PVA-coated strawberries, but strawberries with and without PVA coating can be easily observed to be largely contaminated by molds (Figure 6b). When the strawberries were kept in the fridge, no mold formation was observed on CD/PVA-coated strawberries, while PVA-coated and -uncoated strawberries were contaminated with mold at the end of the 6th day. CD/PVA-coated strawberries were more hydrated and red colored in appearance (Figure 6c).

Further studies were conducted to clarify the effect of the active coating prepared over plane PVA. This time, only half of the strawberries were coated with 0.05GCD/PVA and monitored at RT storage conditions (Figure 6d). As expected, no mold formation was observed on the coated area even after 12 days at RT contrary to the obvious mold population covering the uncoated area on day 4 (see Video S6 and Figure 6d). Our results demonstrated that the CD/PVA coating not only limits mold formation on the fruit but also limits mold spoil through the fruit surface. As compared to the strawberries fully coated with the nanocomposite, semi-coated fruits were dried due to the moisture loss through the uncoated side but stored longer due to decreased water activity (Figure 6d). These results showed that the incorporation of CDs into the coatings and packages not only hinders bacterial activity but also prevents the food to be affected by mold growth, possible spoilage, and moisture loss during storage. The lifetime of perishable foods and fruits such as strawberries could be extended by coating them with the nanocomposite in solution form or storing them in packages made up of these films, and this material could have great potential to create greener and more cost-effective solutions to prevent diseases and economic losses due to food spoilage.

## 4 Conclusions

The current study demonstrated that multicolor-emitting carbon dot-embedded PVA nanocoatings exerted higher thermal stability, UV blocking properties together with considerable antimicrobial activity against important bacterial species, and no cytotoxic activity on MRC-F cells. CD/PVA composites showed a mild antioxidant action involving several antioxidant mechanisms, including metal-ion chelating, radical scavenging, and DNA cleavage activity together with improved anti-adhesive activity against biofilm formation as compared to the plane PVA films. Moreover, their potential as the antifungal coating was shown on strawberries as the model for perishable fruits. CD/PVA coating improved the shelf life by minimizing the fungal growth and spoiling, and moisture loss at both room temperature and fridge conditions. The results offer the possible use of fluorescent CDs as a potential antioxidant and antimicrobial as well as anti-adhesive additive for developing UV-blocking coatings and packages for wrapping perishable foods, which their use could also be extended to the health and pharmaceutical industry.

## Supporting Information Available

The method used for calculating the quantum yield of synthesized CDs, digital images of CD/PVA solutions before the film formation; graphs and tables of quantum yield calculations; Tauc plot diagram of CDs; ^1^H NMR spectra of multicolor CDs; fluorescence emission graphs of PVA and CD/PVA nanocomposite films; FE-SEM images of PVA and CDs/PVA nanocomposite films and TEM images of CDs; XPS survey spectra and high-resolution C 1s, N 1s, and O 1s XPS spectra of CDs; FT-IR and XRD spectra of the synthesized CDs, PVA, and CDs/PVA nanocomposite films; TGA curves of the synthesized PVA and CD/PVA nanocomposite films; digital images showing the visual appearance of uncoated and PVA and 1CD/PVA coated strawberries stored at (a) room and (b) fridge temperature over a time period; tables representing the percentage weight composition of CD/PVA films and effect of CDs on the thermal stability of PVA film (PDF)
Transparency video of BCD-PVA films (MP4)
Transparency video of GCD-PVA films (MP4)
Transparency video of YCD-PVA films (MP4)
Transparency video of RCD-PVA films (MP4)
Strawberry dip coating video (MP4)
Fungal growth on one uncoated strawberry half versus the other half coated with 0.05 GCD/PVA video (MP4)
