# Supplementary material for: Multicolor Emitting Carbon Dot-Reinforced PVA Composites as Edible Food Packaging Films and Coatings with Antimicrobial and UV-Blocking Properties
Source: ACS Omega. 2022 Aug 22;7(34):29967–83. doi: 10.1021/acsomega.2c02984 (PMC9434783; doi:10.1021/acsomega.2c02984)
Supplement: Supplementary file 1 — ao2c02984_si_001.pdf [file ao2c02984_si_001.pdf]

Electronic Supplementary Information (ESI)

Multi-Color Emitting Carbon Dot Reinforced PVA Composites as Edible Food Packaging Films and Coatings with Antimicrobial and UV-Blocking Properties

Melis Özge Alaş[1], Gamze Doğan[2], Mustafa Serkan Yalcin[3], Sadin Ozdemir[4], Rükan Genç[1,5]*

[1] *Department of Chemical Engineering, Engineering Faculty, Mersin University, TR-33343, Mersin, Turkey*

[2] *Izmir Institute of Technology Faculty of Engineering Department of Bioengineering, TR-35430, Urla-Izmir, Turkey*

[3] *Department of Chemistry and Chemical Processing Technologies, Technical Science Vocational School, Mersin University, TR-33343 Yenisehir, Mersin, TURKEY*

[4] *Food Processing Programme, Technical Science Vocational School, Mersin University, TR-33343 Yenisehir, Mersin, Turkey*

[5] *Sabanci University, Nanotechnology Research and Application Centre, TR-34956, Istanbul, Turkey*

**Corresponding author: rukan.genc@sabanciuniv.edu*

Quantum Yield Calculation of multi-color CDs

Rhodamine B in water (quantum yield in literature 0.31) was chosen as the standard [1,2]. The quantum yield (QY) of CDs in water was calculated according to the following equation:

$$\Phi_{x} = \Phi_{ST}\left(\frac{\mathrm{Grad}_{X}}{\mathrm{Grad}_{ST}}\right)\left(\frac{\eta_x^2}{\eta_{ST}^2}\right)$$

Where Φ is the quantum yield, grad measured slope, n is the refractive index (1.33 for water). The subscript ST refers to the reference fluorophore of known QY. In order to obtain more credible results, the concentrations of BCD and reference fluorescent dyes were adjusted so that the optical absorbance values at 365 nm are between 0 and 0.1. The PL spectra were measured, and the PL intensity was integrated. QYs were defined with a comparison of the integrated PL intensity and absorbance curves (Figure S2-S5).

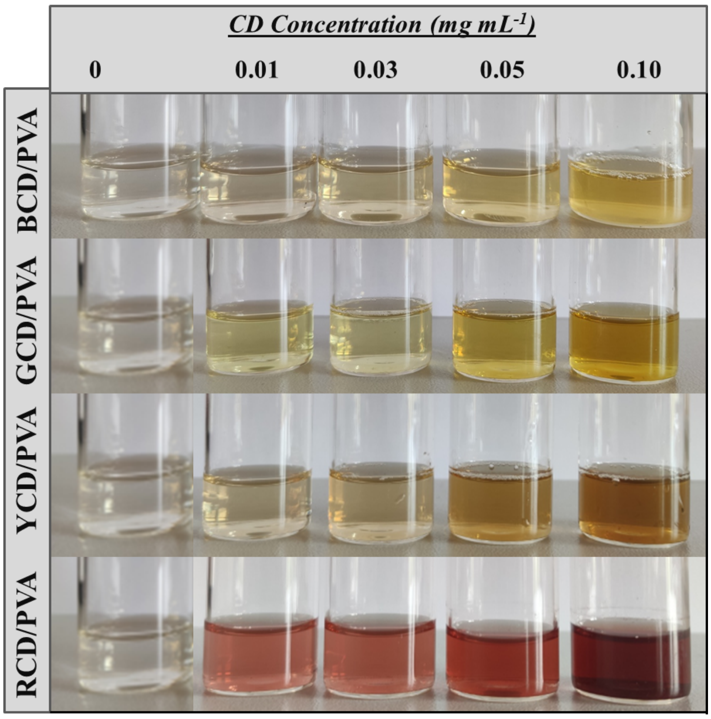

Figure S1. Digital images of CD / PVA mixtures with varying CD concentrations (0-0.1 w/w %)

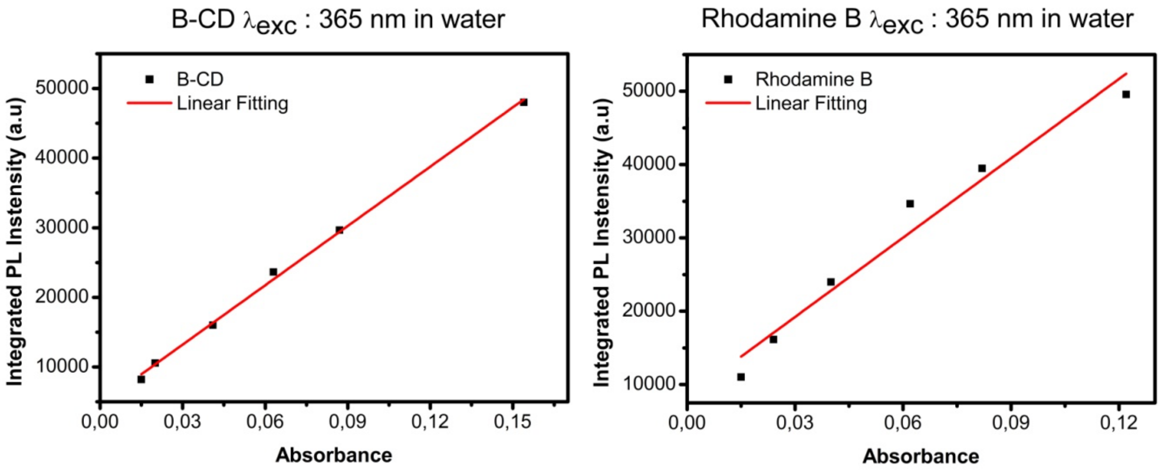

	BCD						Rhodamine B					
**Abs**	0.015	0.020	0.041	0.063	0.087	0.154	0.015	0.024	0.040	0.062	0.082	0.122
**Integrated PL**	8213	10568	16015	23648	29654	48000	11028	16153	23971	34648	39467	49559
**Slope**	284094.5						360562.4					

**Ref. index**	1.33	1.33
**QY**	**24.4%**	31%

Figure S2. Plots of integrated fluorescence intensity against the absorbance of B-CD and Rhodamine B (referenced dye) at 365 nm.

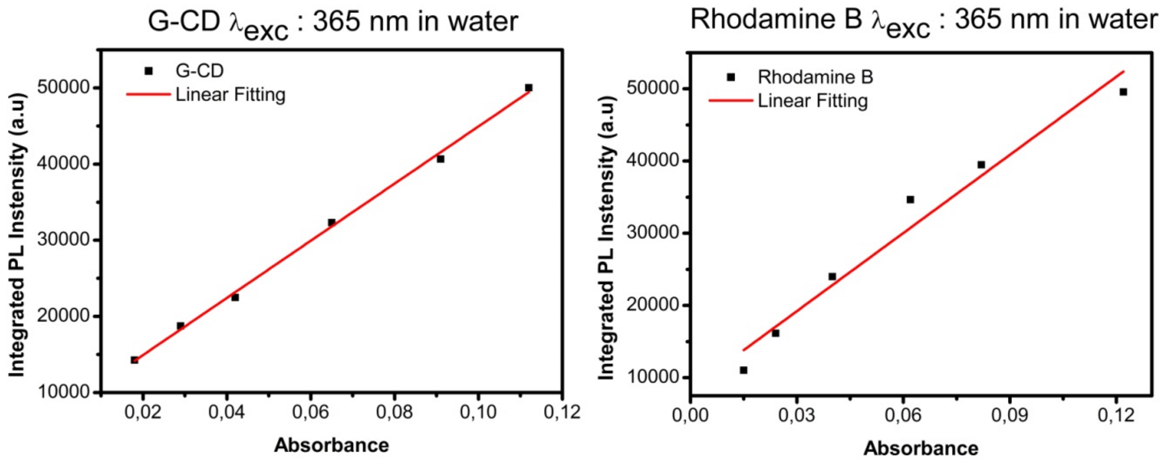

	**GCD**						**Rhodamine B**					
**Abs**	0.018	0.029	0.042	0.065	0.091	0.112	0.015	0.024	0.040	0.062	0.082	0.122
**Integrated PL**	14260	18731	22489	32312	40646	50033	11028	16153	23971	34648	39467	49559
**Slope**	375237.2						360562.4					
**Ref. index**	1.33						1.33					
**QY**	**32.3%**						31%					

Figure S3. Plots of integrated fluorescence intensity against the absorbance of G-CD and Rhodamine B (referenced dye) at 365 nm.

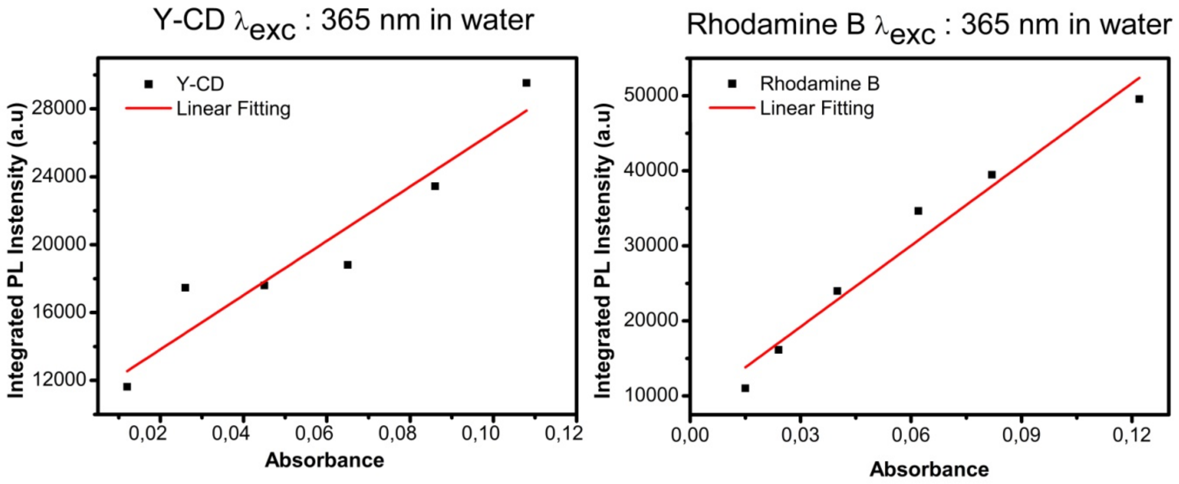

	YCD						Rhodamine B					
**Abs**	0.012	0.026	0.045	0.065	0.086	0.108	0.015	0.024	0.040	0.062	0.082	0.122
**Integrated PL**	11625	17468	17590	18810	23441	29529	11028	16153	23971	34648	39467	49559
**Slope**	159820.8						360562.4					
**Ref. index**	1.33						1.33					
**QY**	**13.7%**						31%					

Figure S4. Plots of integrated fluorescence intensity against the absorbance of Y-CD and Rhodamine B (referenced dye) at 365 nm.

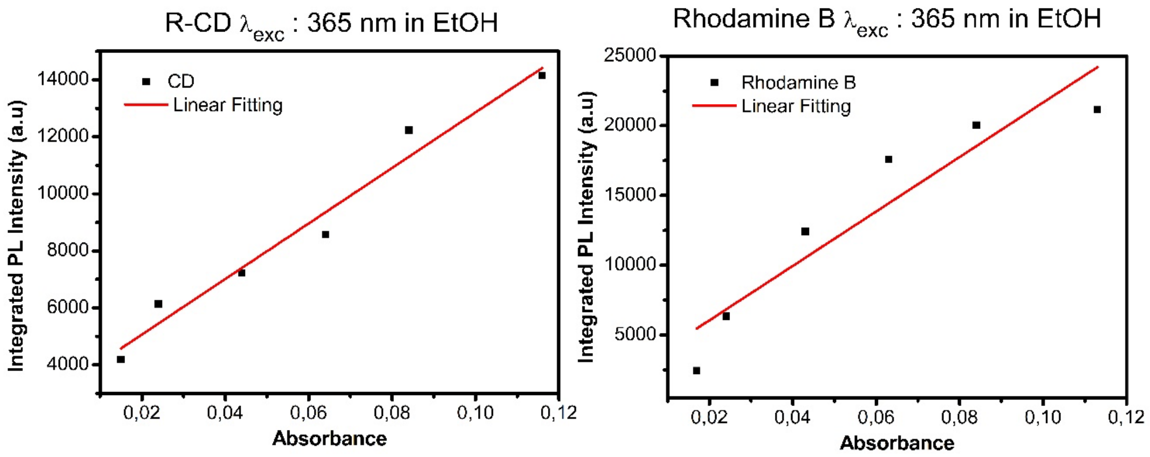

	RCD						Rhodamine B					
**Abs**	0.015	0.024	0.044	0.064	0.084	0.116	0.017	0.024	0.043	0.063	0.084	0.114
**Integrated PL**	14150	12235	8579	7215	6139	4185	21178	20045	17587	12421	6352	2451
**Slope**	97472.6						195198.9					
**Ref. index**	1.36						1.33					
**QY**	**29.2%**						56%					

Figure S5. Plots of integrated fluorescence intensity against the absorbance of RCD and Rhodamine B (referenced dye) at 365 nm.

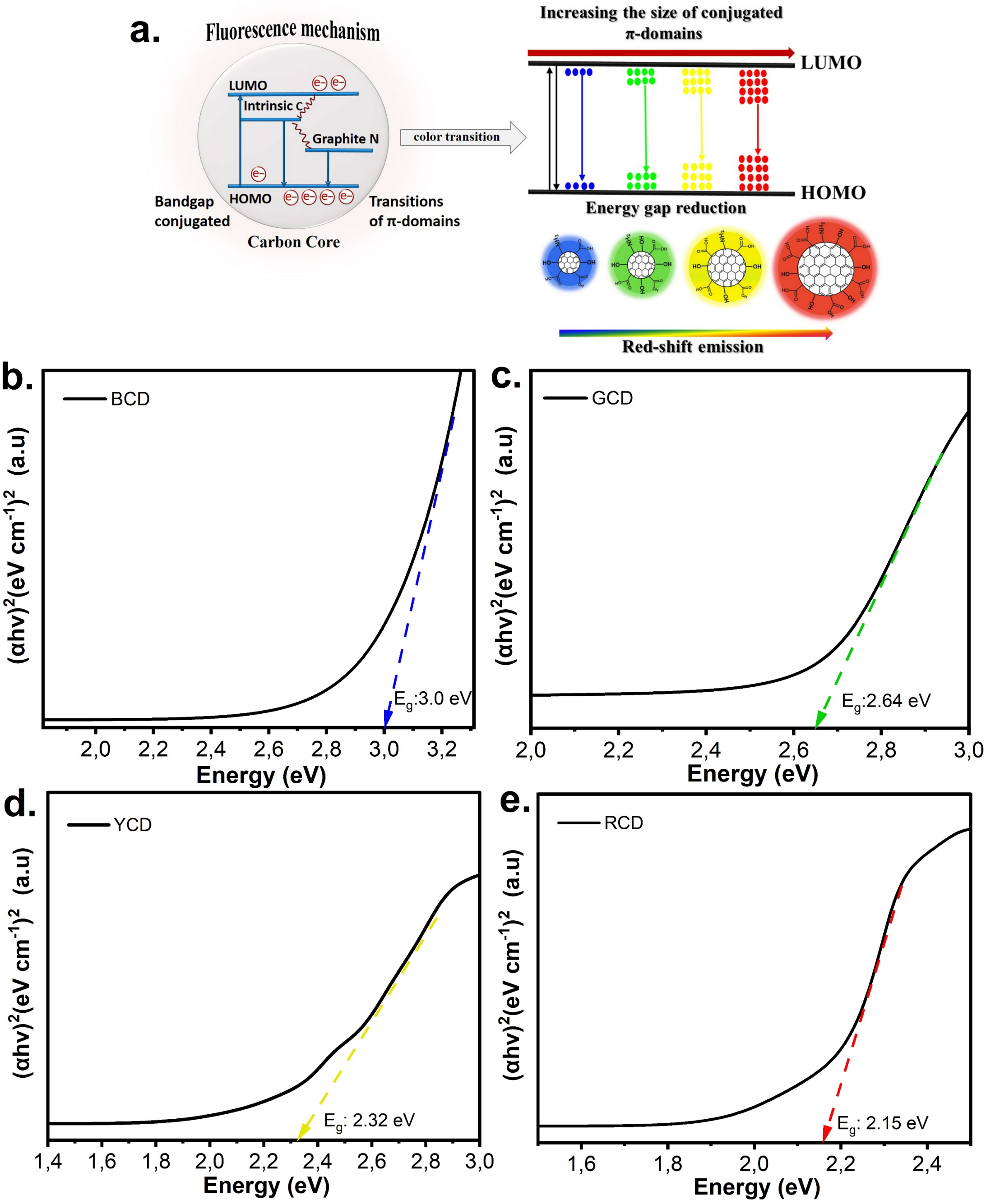

Figure S6. (a) A scheme of electronic bandgap transitions structure of conjugated π-domains and possible fluorescence color for CDs, Tauc plot diagramı of (b) BCD, (c) GCD, (d) YCD, and (e) RCD.

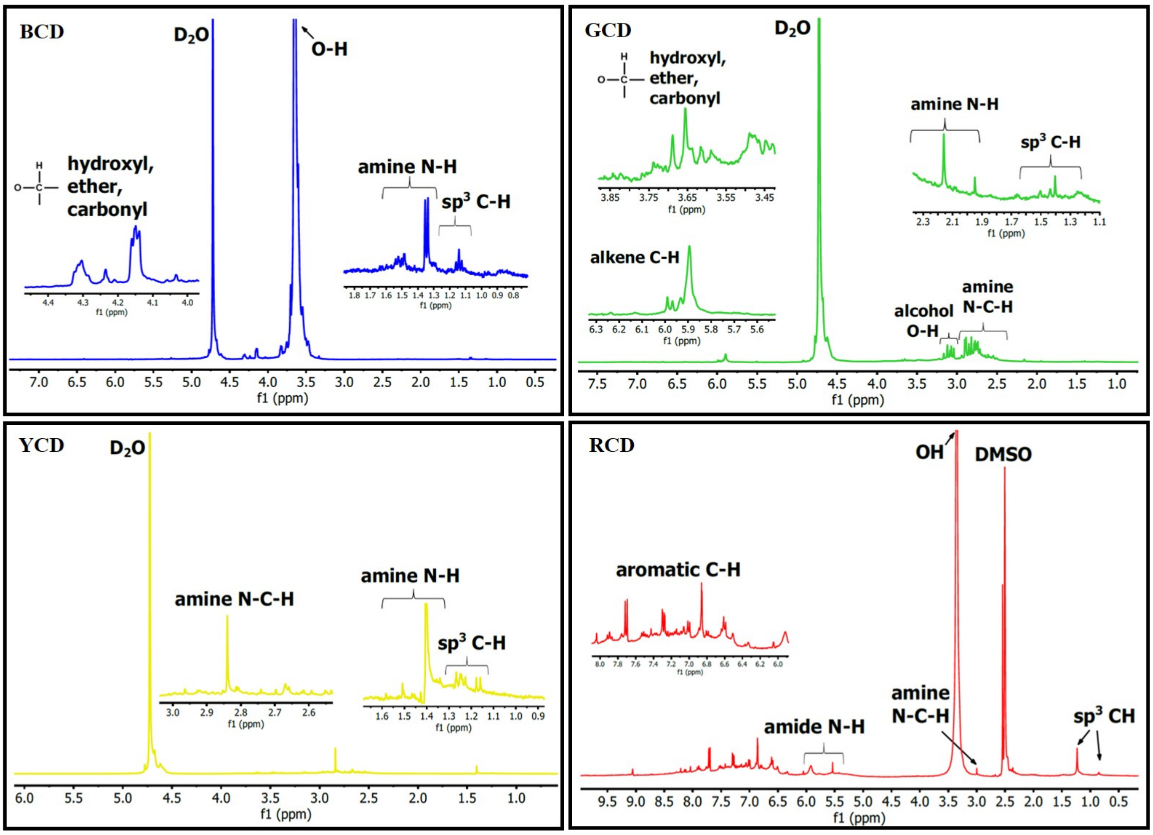

Figure S7. ^{1}H NMR spectra of multi-color CDs.

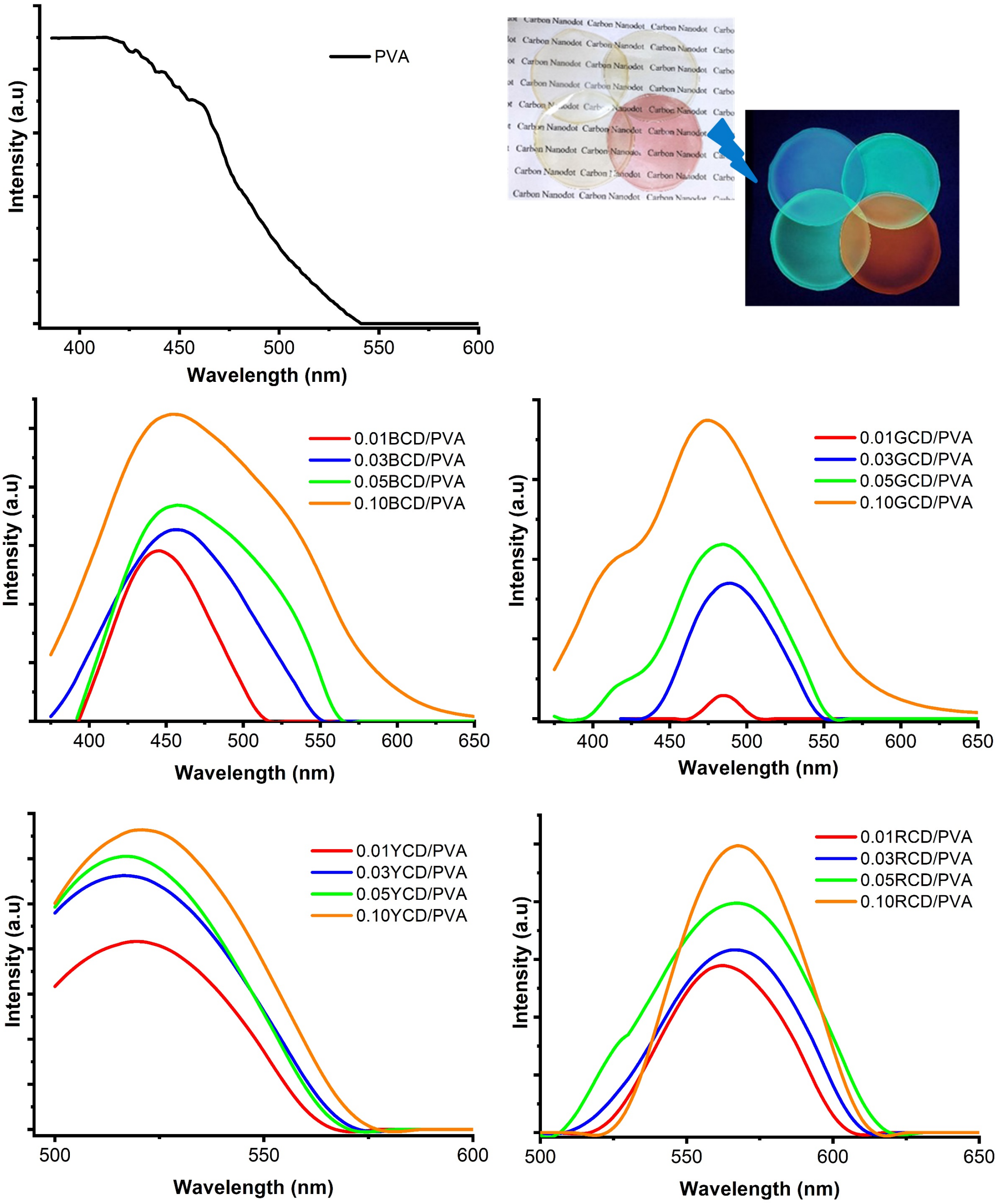

Figure S8. Fluorescence emission graphs of PVA and CD/PVA nanocomposite films made up of varying CD concentrations (0.01-0.1 w/w % CD/PVA).

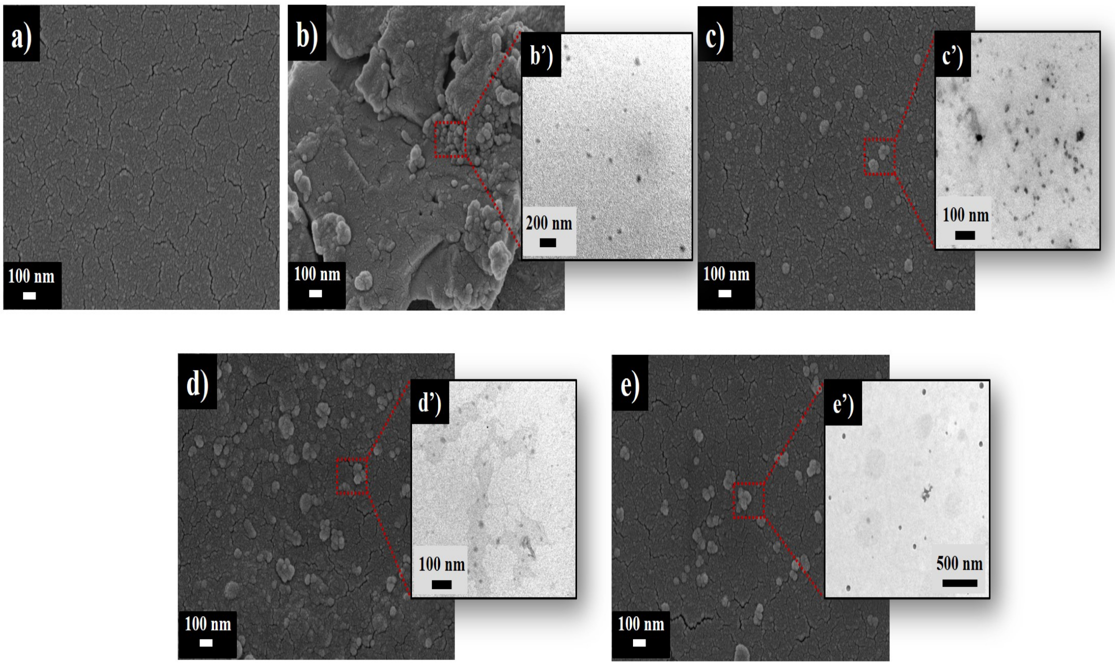

Figure S9. FE-SEM images of (a) PVA, (b) 0.05BCD / PVA, (c) 0.05GCD / PVA, (d) 0.05YCD / PVA, and (e) 0.05RCD / PVA nanocomposite films, TEM images of (b') BCD, (c') GCD, (d') YCD, and (e') RCD.

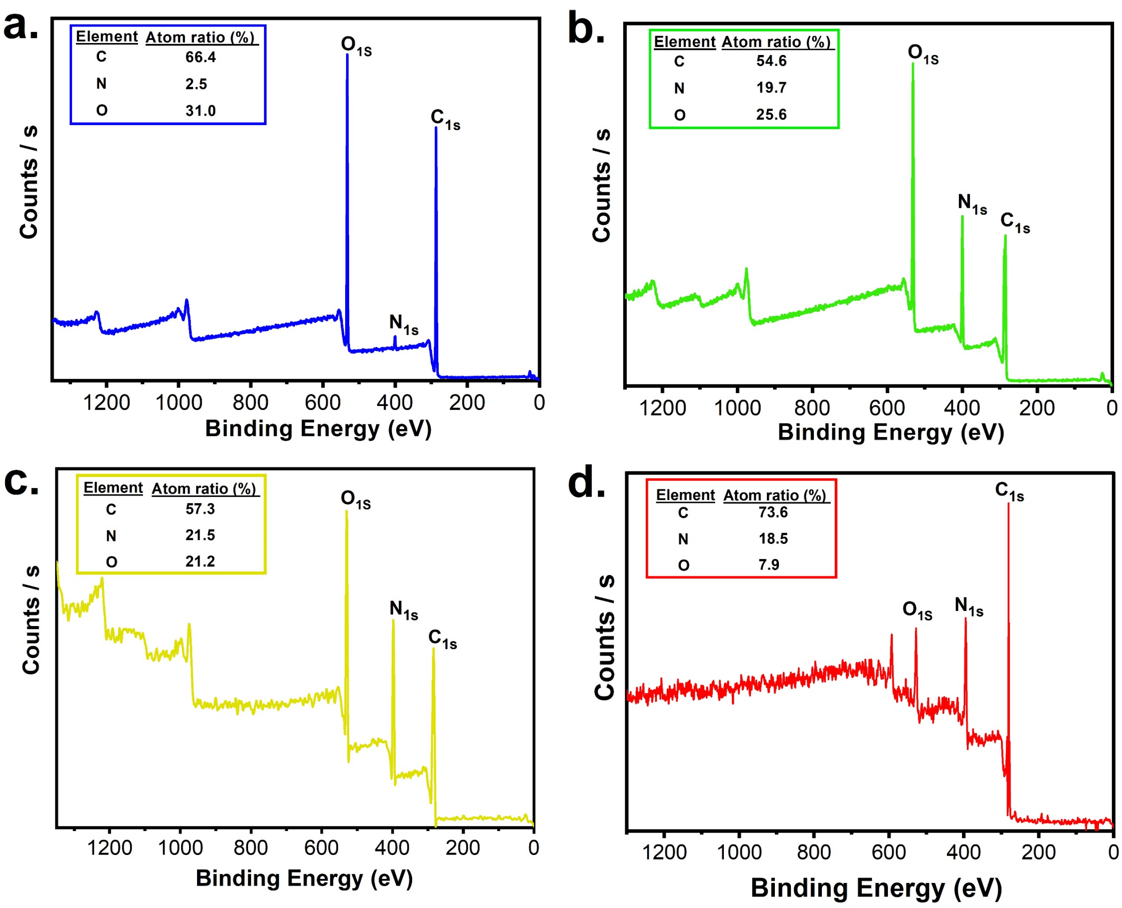

Figure S10. XPS survey spectra of (a) BCD, (b) GCD, (c) YCD, and (d) RCD (inset: the table giving the atomic ratios (%) of the elements).

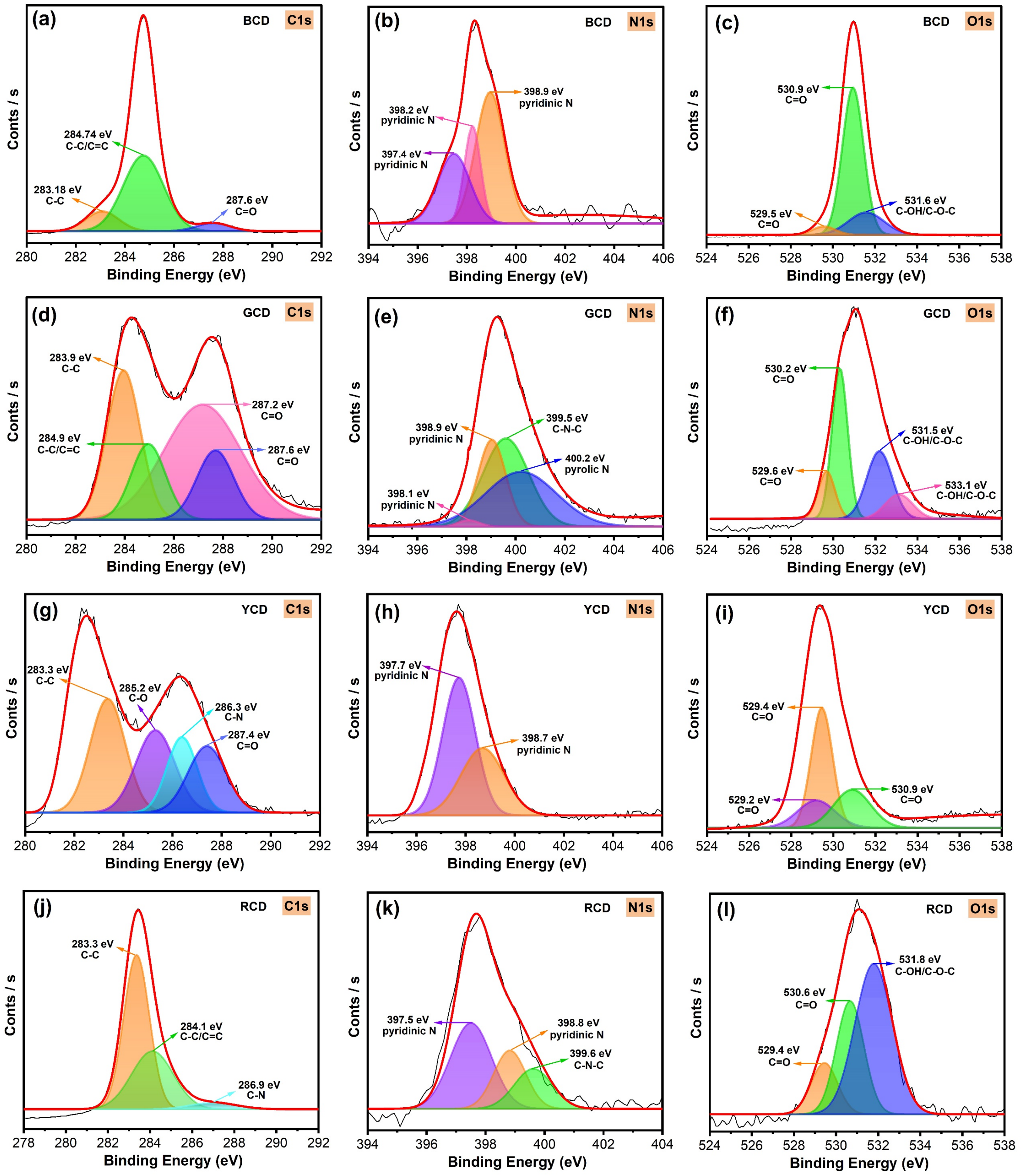

Figure S11. High-resolution XPS spectra of C1s, N1s, and O1s of (a-c) BCD, (d-f) GCD, (g-i) YCD, and (j-l) RCD.

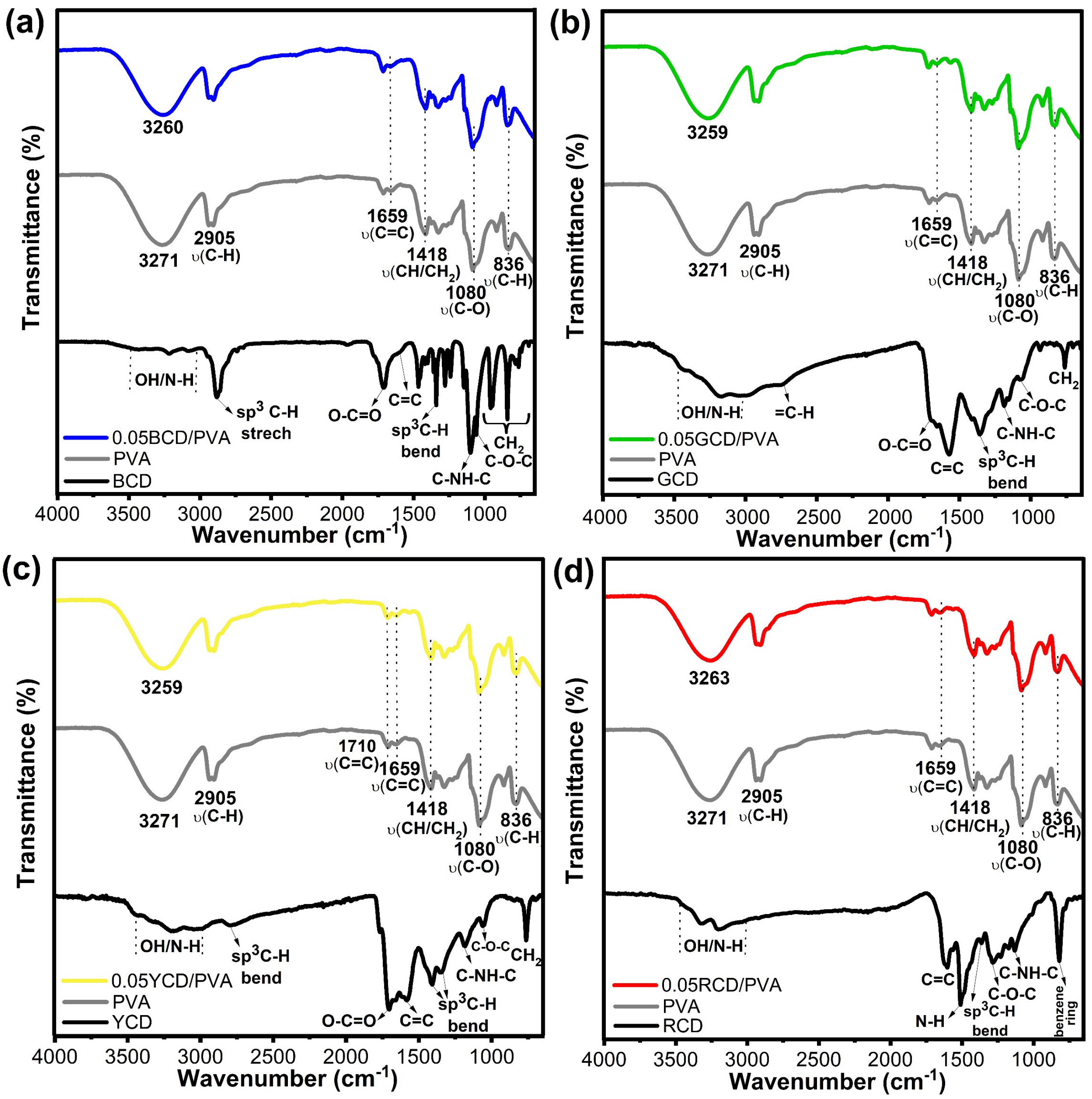

Figure S12. FT-IR spectrums of (a) BCD, PVA and 0.05BCD/PVA films, (b) GCD, PVA and 0.05 GCD/PVA films, (c) YCD, PVA and 0.05YCD/PVA films, and (d) RCD, PVA and 0.05RCD/ PVA films.

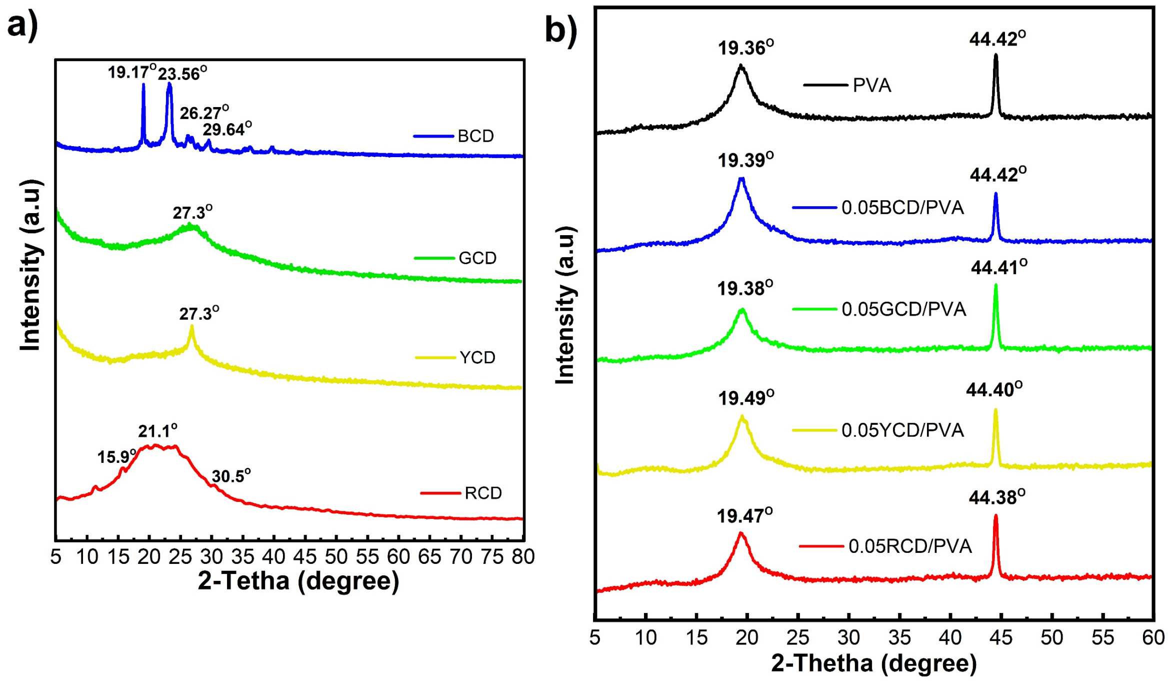

Figure S13. XRD spectra of (a) multi-color CDs and (b) comparison of PVA and 0.05CD/PVA films emitting different color.

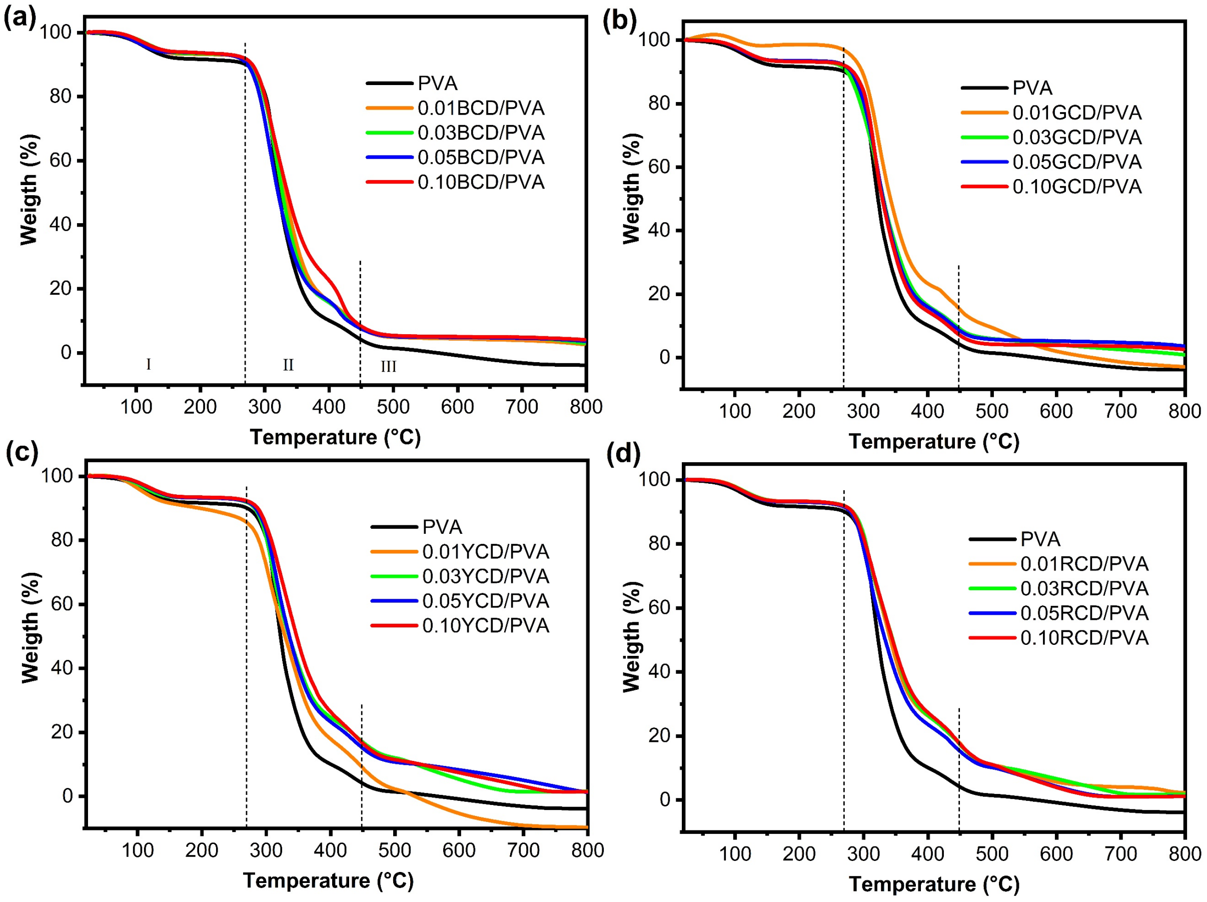

Figure S14. TGA curves of the synthesized (a) PVA and BCD/PVA films, (b) PVA and GCD/PVA films, (c) PVA and YCD/PVA films, and (d) PVA and RCD/PVA films (CD was integrated into PVA at varying concentrations (0.01-0.1 w/w %)

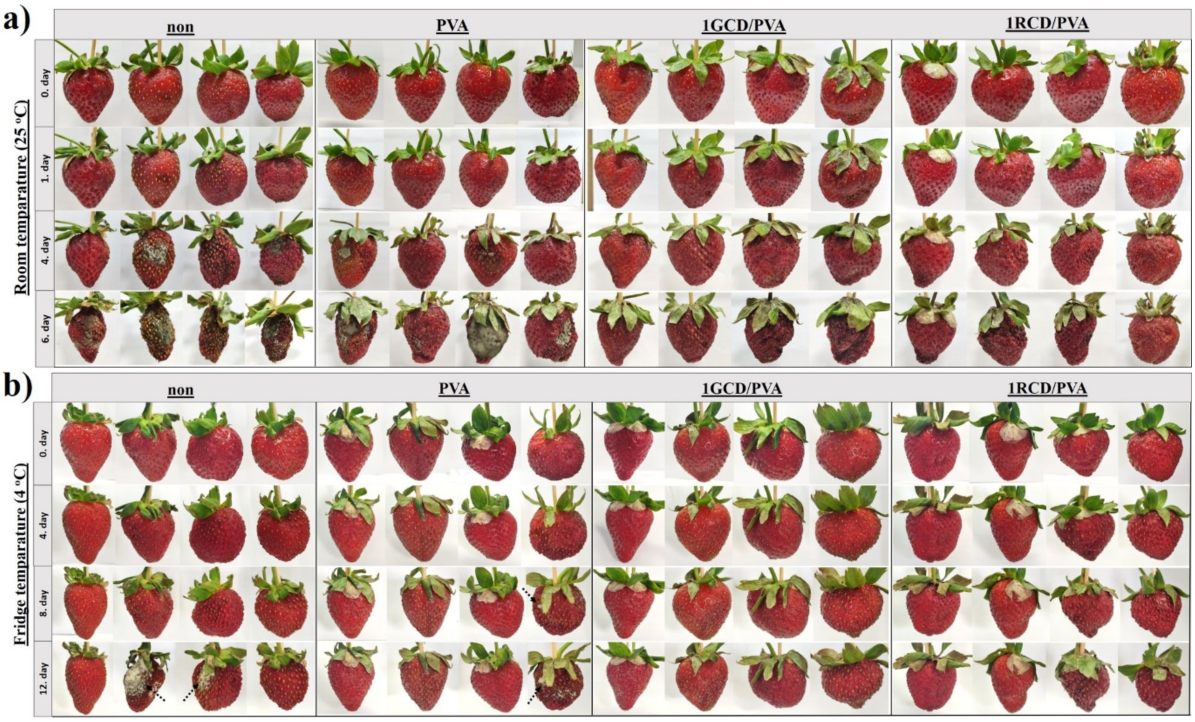

Figure S15. Digital images of the appearance of non-coated and coated strawberries with PVA and 0.05CD/PVA films at (a) room, and (b) fridge temperature during storage times.

Table S1. The percentage weight composition of CD/PVA films produced.

Samples	PVA Concentration (mg mL^{-1})	CD Concentration (mg mL^{-1})	Weight percentage of CDs in CD/PVA film (w/w %)
PVA	75.00	0.00	0.00
0.01CD/PVA	75.00	0.01	0.02
0.03CD/PVA	75.00	0.03	0.03
0.05CD/PVA	75.00	0.05	0.07
0.10CD/PVA	75.00	0.10	0.13

Table S2. Effect of CD incorporation on the thermal stability of PVA film.

Samples	T_{50}	Weight loss at T_{max} (%)	Residue at 800°C

	**(ºC)**		**(wt %)**
PVA	322.5	100.00	0.00
0.01BCD/PVA	331.8	97.33	2.67
0.03BCD/PVA	327.3	96.95	3.05
0.05BCD/PVA	322.6	96.32	3.68
0.10BCD/PVA	335.4	95.86	4.14
0.01GCD/PVA	342.0	99.00	1.00
0.03GCD/PVA	331.2	99.07	1.03
0.05BCD/PVA	330.6	96.38	3.62
0.10BCD/PVA	329.3	97.35	2.65
0.01YCD/PVA	329.3	100.00	0.00
0.03YCD/PVA	335.6	98.39	1.61
0.05YCD/PVA	336.0	98.66	1.34
0.10YCD/PVA	347.3	98.49	1.51
0.01RCD/PVA	341.8	97.64	2.36
0.03RCD/PVA	345.2	98.22	1.78
0.05RCD/PVA	333.5	98.82	1.18
0.10RCD/PVA	344.8	98.83	1.17

References

(1) Zong, J.; Zhu, Y.; Yang, X.; Shen, J.; Li, C. Synthesis of Photoluminescent Carbogenic Dots Using Mesoporous Silica Spheres as Nanoreactors. *Chem. Commun* **2011**, *47* (2), 764–766.

(2) Shi, Y.; Li, C.; Liu, S.; Liu, Z.; J., Z.; Yang, J.; Hu, X. Facile Synthesis of Fluorescent Carbon Dots for Determination of Curcumin Based on Fluorescence Resonance Energy Transfer. *RSC Adv.* **2015**, *5* (79), 64790–64796.
